# A flexible liposomal polymer complex as a platform of specific and regulable immune regulation for individual cancer immunotherapy

**DOI:** 10.1186/s13046-023-02601-8

**Published:** 2023-01-23

**Authors:** Chia-Hung Chen, Tzu-Han Weng, Hsiao-Hsuan Huang, Ling-Ya Huang, Kai-Yao Huang, Pin-Rong Chen, Kuang-Yu Yeh, Chi-Ting Huang, Yu-Tzu Chien, Po-Ya Chuang, Yu-Ling Lin, Nu-Man Tsai, Shih-Jen Liu, Yu-Cheng Su, Shun-Long Weng, Kuang-Wen Liao

**Affiliations:** 1 Department of Medical Research, Hsinchu MacKay Memorial Hospital, Hsinchu City, 30071 Taiwan; 2 Department of Dermatology, MacKay Memorial Hospital, Taipei City, 10449 Taiwan; 3 Industrial Development Graduate Program of College of Biological Science and Technology, National Yang Ming Chiao Tung University, Hsinchu City, 30068 Taiwan; 4 Institute of Bioinformatics and Systems Biology, National Yang Ming Chiao Tung University, Hsinchu City, 30068 Taiwan; 5 Department of Medicine, MacKay Medical College, 25245 New Taipei City, Taiwan; 6 Institute of Molecular Medicine and Bioengineering, National Yang Ming Chiao Tung University, Hsinchu City, 30068 Taiwan; 7 Department of Biological Science and Technology, National Yang Ming Chiao Tung University, 30068 Hsinchu City, Taiwan; 8 Agricultural Biotechnology Research Center, Academia Sinica, Taipei, 11529 Taiwan; 9 Department of Medical Laboratory and Biotechnology, Chung Shan Medical University, Taichung City, 40201 Taiwan; 10 Department of Pathology and Clinical Laboratory, Chung Shan Medical University Hospital, Taichung City, 40201 Taiwan; 11 National Institute of Infectious Diseases and Vaccinology, National Health Research Institutes, 350401 Miaoli, Taiwan; 12 Department of Obstetrics and Gynecology, Hsinchu MacKay Memorial Hospital, Hsinchu City, 30071 Taiwan; 13 MacKay Junior College of Medicine, Nursing and Management, Taipei City, 11260 Taiwan; 14 Graduate Institute of Medicine, College of Medicine, Kaohsiung Medical University, Kaohsiung City, 80708 Taiwan; 15 College of Dental Medicine, Kaohsiung Medical University School of Dentistry, Kaohsiung City, 80708 Taiwan; 16 Department of Biotechnology and Bioindustry Sciences, National Cheng Kung University, Tainan City, 70101 Taiwan; 17 Center for Intelligent Drug Systems and Smart Bio-Devices, National Yang Ming Chiao Tung University, Hsinchu City, 30068 Taiwan

**Keywords:** LPPC, Immunotherapy, Costimulatory molecules, personalized cancer therapy, Tumor heterogenicity

## Abstract

**Background:**

The applicability and therapeutic efficacy of specific personalized immunotherapy for cancer patients is limited by the genetic diversity of the host or the tumor. Side-effects such as immune-related adverse events (IRAEs) derived from the administration of immunotherapy have also been observed. Therefore, regulatory immunotherapy is required for cancer patients and should be developed.

**Methods:**

The cationic lipo-PEG-PEI complex (LPPC) can stably and irreplaceably adsorb various proteins on its surface without covalent linkage, and the bound proteins maintain their original functions. In this study, LPPC was developed as an immunoregulatory platform for personalized immunotherapy for tumors to address the barriers related to the heterogenetic characteristics of MHC molecules or tumor associated antigens (TAAs) in the patient population. Here, the immune-suppressive and highly metastatic melanoma, B16F10 cells were used to examine the effects of this platform. Adsorption of anti-CD3 antibodies, HLA-A2/peptide, or dendritic cells’ membrane proteins (MP) could flexibly provide pan-T-cell responses, specific Th1 responses, or specific Th1 and Th2 responses, depending on the host needs. Furthermore, with regulatory antibodies, the immuno-LPPC complex properly mediated immune responses by adsorbing positive or negative antibodies, such as anti-CD28 or anti-CTLA4 antibodies.

**Results:**

The results clearly showed that treatment with LPPC/MP/CD28 complexes activated specific Th1 and Th2 responses, including cytokine release, CTL and prevented T-cell apoptosis. Moreover, LPPC/MP/CD28 complexes could eliminate metastatic B16F10 melanoma cells in the lung more efficiently than LPPC/MP. Interestingly, the melanoma resistance of mice treated with LPPC/MP/CD28 complexes would be reversed to susceptible after administration with LPPC/MP/CTLA4 complexes. NGS data revealed that LPPC/MP/CD28 complexes could enhance the gene expression of cytokine and chemokine pathways to strengthen immune activation than LPPC/MP, and that LPPC/MP/CTLA4 could abolish the LPPC/MP complex-mediated gene expression back to un-treatment.

**Conclusions:**

Overall, we proved a convenient and flexible immunotherapy platform for developing personalized cancer therapy.

**Graphical Abstract:**

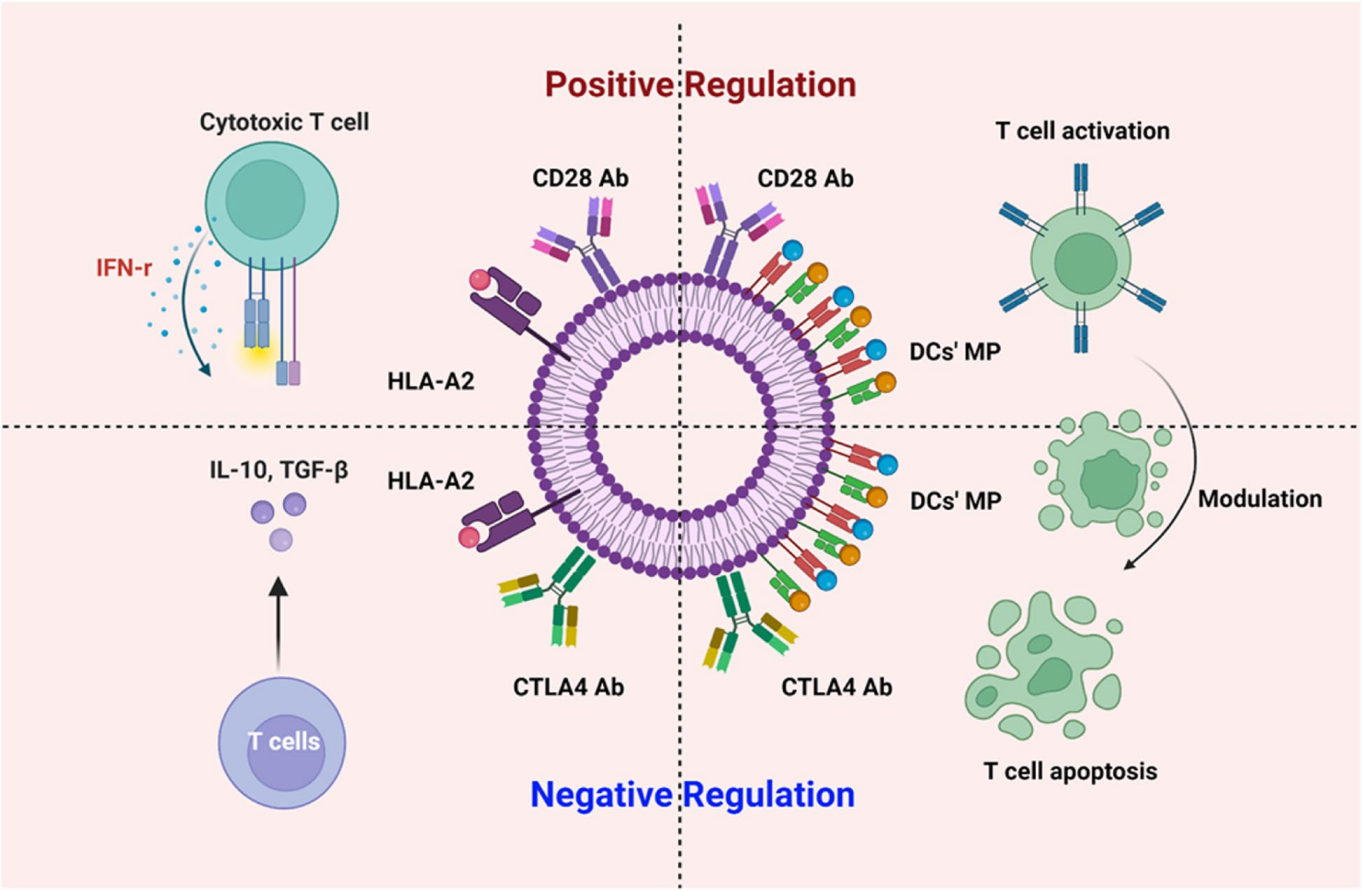

**Supplementary Information:**

The online version contains supplementary material available at 10.1186/s13046-023-02601-8.

## Background

Immunotherapy is the crucial frontier of current personalized cancer therapy. These are the voyages of immunotherapy research. Its current missions: To explore novel individual tumor targets; to seek out new methods and new materials for personalized cancer therapy; and to regulate the pathogenic effect of immunotherapy what is unexpected before. In other words, cancer immunotherapy aims to efficiently eliminate harmful cells, such as metastatic tumor cells, and to protect normal tissues from autoimmune attack [1, 2]. These are closely related to the heterogeneities of the major histocompatibility complex (MHC) and tumor associated antigens (TAAs) in individual cancer patients. For appropriate and efficient immunomodulation of personalized precision therapy, T-cell immune homeostasis should be addressed. T cells are mainly involved in specifically regulating adaptive immunity, and antigen presenting cells (APCs) elicit specific and effective T-cell mediated immunity by antigen-derived peptides of MHC presentation, which has been recognized as the crucial event in this process [3]. However, there is MHC diversity in the human population due to genetic polymorphism. Therefore, it is crucial to incorporate the appropriate MHC molecules into the therapeutic process to trigger an effective antitumor T-cell reaction.

Excluding MHC restriction, chimeric antigen receptor T cells (CAR-T cells) have shown dramatic therapeutic efficacy for patients, but they also have some further challenges and drawbacks to ameliorate [4, 5]. As genetically modified immune cells, specific CAR-T cells are still influenced by suppressive factors in the tumor area; for example, regulatory T cells express IL-10 and TGF-β to suppress overactivated T cells and induce apoptosis in T cells [6, 7]. Therefore, the tumor microenvironment influences the therapeutic efficacy of CAR-T cells. Another challenge for the use of CAR-T cells in personalized cancer therapy is that the expression of TAAs in patients is heterogenic. For example, the well-known oncogene Her-2, is overexpressed in only approximately 25% of patients with breast cancer or gastric cancer [8, 9]. Thus, even if MHC restriction were resolved, most cancer patients still could not obtain a benefit from this immunotherapy.

Without the abovementioned difficulty, immune checkpoint inhibitor (ICI) therapy can efficiently decrease tumor growth, such as anti-CTLA4 or anti-PDL1 antibodies [10]. However, many studies have reported that ICIs increase the risk of autoimmune disease to induce immune-related adverse events (IRAEs) [11]. One therapeutic dilemma is that higher toxicity of IRAEs leads to a better objective response rate but worse overall survival, as shown by a meta-analysis [12]. At present, nonspecific immunomodulator glucocorticoids are used to rescue patient symptoms related to IRAEs, but they cause other systemic adverse side effects [13]. Therefore, precise immunoregulation is needed for proper immunotherapy to prevent IRAEs and other side effects.

Lipo-PEG-PEI complex (LPPC) could stably adsorb proteins via noncovalent linkages and retain their activities on the surface, both in vitro and in vivo [14]. In addition, LPPC encapsulating antitumor drugs could increase their antitumor effects, even abolishing drug-resistance [15, 16]. In terms of its applicability for unusual in vivo delivery routes, LPPC was applied to protein intercellular delivery for enzyme-defect therapy [17], efficient drug delivery across the BBB for brain tumor suppression and transdermal drug delivery for breast cancer inhibition [18, 19]. Based on such characteristics, the LPPC complex could efficiently target tumors by targeting antibodies and deliver therapeutic drugs or transgenes, resulting in dramatic therapeutic efficacy, even achieving total cure (100% tumor-free) in an animal model of human breast tumor [20, 21]. Therefore, this study attempts to address the unmet needs described above by utilizing autologous dendritic cell membrane proteins (DC MP), which highly expressing MHC and pulsing protein antigens [3] (Additional file 1, upper). In addition, antibodies (Abs) interact as the ligand and receptor of APCs and T cells at the immune synapse that have been developed [22, 23]. Therefore, the DC MP would combine with the immunoregulatory monoclonal antibodies (mAbs) to adsorb onto LPPC to form LPPC/MP/mAb complexes for regulation of the specific T-cell immune responses (Additional file 1, lower). In this study, we developed an immunoregulation platform which utilized LPPC stably adsorbing DC MP and anti-costimulatory Abs to regulate specific immunity positively or negatively in the metastatic tumor model. The study demonstrated that LPPC was a critical and easy-manipulated vector for activating specific T-cell responses by DC MP and efficiently regulating immune responses by anti-costimulatory Abs. Therefore, this platform will be developed further for personalized cancer therapy to eliminate metastatic tumor cells and reverse IRAEs.

## Methods

### Reagents and antibodies

1,2-Dioleoyl-*sn*-glycero-3-phosphocholine (DOPC) and 1,2-dilauroyl-*sn*-glycero-3-phosphocholine (DLPC) were purchased from Avanti Polar Lipids (Alabaster, AL, USA). Polyethylene glycol (PEG, MW 8,000), polyethyleneimine (PEI branched, MW 25,000), Complete Freund’s adjuvant (CFA), Incomplete Freund’s adjuvant (IFA), and methylthiazolyldiphenyl-tetrazolium bromide (MTT) were purchased from Sigma‒Aldrich (St. Louis, MO, USA). Anti-mouse CTLA4 monoclonal antibody (mAb) and anti-mouse CD28 mAb were purchased from BioLegend (San Diego, CA, USA). Anti-human CD3 mAb (OKT3) and anti-mouse CD3 mAb (2C11) were kindly provided by Dr. Steve R. Roffler (Institute of BioMedical Sciences, Academia Sinica, Taipei City, Taiwan, ROC).

### Others

Peptide-HLA-A2 monomer and one identify epitope of HPV type 16 E7 protein (YMLDLQPETT) were kindly provided by Dr. Shih-Jen Liu (NHRI) [24]. Additionally, TC1/AAD cells were gifts from Dr. Shih-Jen Liu (NHRI).

### Analysis of LPPC size and surface charge

LPPCs were prepared according to the procedures outlined in previous studies [14, 20]. The particle size and the surface charge of LPPC were analyzed by a Zetasizer (Zetasizer Nano ZS90, Malvern, UK). Briefly, ten micrograms of LPPC were complexed with or without immune molecules, such as 0.15 µg anti-CD28 mAb, 0.15 µg anti-CTLA4 mAb, 20 µg dendritic cells membrane proteins or 0.6 µg HLA-A2 protein. After adsorption, the addition of PEG into immuno-LPPC complexes neutralized the amine group of PEI from the LPPC components. The different combinations of immuno-LPPC complexes were listed in Table 1. LPPC complexes were applied onto a glow-discharged TEM grid (300-mesh Cu grid) coated with a holey carbon film. The excess liquid was blotted, and the specimens were vitrified by rapid plunging into liquid ethane precooled with liquid nitrogen. Cryo-TEM was performed on a CEM2 instrument (Tecnai G2 F20 TWIN) at Academia Sinica, Taiwan.

**Table 1:** The particle sizes and surface charges of LPPC in combination with immunofunctional molecules. LPPC adsorbed different mAbs and proteins in the following experiments. The addition of PEG for charge neutralization was represented as w/, otherwise, it was shown as w/o. A Zetasizer was used to analyze the characteristics, particle size, and zeta-potential of LPPC with different molecules. All values were the mean ± SD of three independent experiments (*N* = 6)

Formulation	Size (nm)	Zeta-potential (mV)
LPPC (w/o)	**184.2 ± 12.4**	**40.7 ± 5.1**
LPPC (w/)	**208.4 ± 19.2**	**8.2 ± 4.5**
LPPC/OKT3/CD28 (w/)	**221.7 ± 13.1**	**11.8 ± 4.1**
LPPC/2C11/CD28 (w/)	**214.3 ± 21.2**	**9.7 ± 1.5**
LPPC/MP (w/)	**255.9 ± 18.2**	**8.1 ± 3.1**
LPPC/MP/CD28 (w/)	**275.0 ± 21.6**	**6.9 ± 4.4**
LPPC/MP/CTLA4 (w/)	**264.1 ± 15.7**	**5.8 ± 2.9**
LPPC/HLA/CD28 (w/)	**325.6 ± 25.1**	**10.8 ± 4.4**
LPPC/HLA/CTLA4 (w/)	**314.1 ± 19.5**	**8.4 ± 3.6**

The data represent the mean ± SD (*N* = 6)

### The capacity and activity of LPPC with mAbs

Ten micrograms of LPPC were incubated with different amounts of OKT3 or 2C11 for 20 min and centrifuged at 5,900 × g for 5 min. The pellets were resuspended, and the quantity of OKT3 or 2C11 bound to LPPC was determined by using a Coomassie Plus Bradford Assay Kit (Sigma‒Aldrich) according to the standard procedures provided by the manufacturer. The supernatants were analyzed as described above.

Peripheral blood mononuclear cells (PBMCs) were seeded in a 96-well plate at 1 × 10^5^ per well in 100 µl RPMI medium containing 10% FBS and 1% PSA. LPPCs (1µγ) with different amounts of mAbs were added respectively to each well of a 96-well plate. After incubation for three days, the cell proliferation rates were determined by the MTT assay (Sigma‒Aldrich).

Similarly, in the naive group, splenocytes (2.5 × 10^5^ cells, each well of a 96-well plate) from naive mice were incubated with 1 µg LPPC with different amounts of mAbs for three days. In the activated groups, splenocytes from naive mice were activated by anti-CD3 (60 ng/100 µl) and anti-CD28 mAbs (60 ng/100 µl) for three days. After stimulation, the splenocytes were reseeded into a 96-well plate. The cell proliferation rate was monitored by MTT assay.

### Real-time PCR

Naive or activated splenocytes prepared as described above were incubated with LPPC, LPPC/CD3, LPPC/CD3/CD28, or LPPC/CD3/CTLA4 for 12 h, and approximately 1 × 10^6^ cells were centrifuged at 400 × g for 5 min. Then, total RNA was extracted from the cell pellets using TRIzol reagent (Invitrogen, Gaithersburg, MD, USA) following the manufacturer's instructions.

All real-time PCR reactions were performed using an ABI Prism 7000 Sequence Detection System (Perkin-Elmer Applied Biosystems, Foster City, CA, USA). The 2(-ΔΔ)(CT) method was used to calculate relative changes in gene expression as determined by real-time quantitative PCR experiments. In the present study, the data are presented as the fold change in target gene (IL-2) expression in splenocytes normalized to the internal control gene (β-actin).

### Caspase 3 activity

Naive or activated splenocytes were incubated respectively with different treatments for 4 h, and then the caspase 3 activity of the cells was determined by the PE-active caspase 3 apoptosis kit (Becton Dickinson, Mountain View, CA, USA) and analyzed by flow cytometry.

### Cell cycle

Naive or activated splenocytes were incubated with different treatments for 48 h, as in a previous study [15], the 1 × 10^6^ cell pellets were washed with PBS and fixed in 3 ml of 70% ethanol at 4 °C for 30 min. After centrifugation at 400 × g, the fixed cells were resuspended in 1 ml of DNA staining buffer (containing 5% Triton-X 100, 0.1 mg/ml RNase A and 4 μg/ml propidium iodide) and incubated for 30 min at room temperature. Ten thousand cells were analyzed for DNA content using a Becton–Dickinson FACScan (Becton Dickinson, Mountain View, CA), and the cell cycle distribution was determined using ModFit software (Becton Dickinson).

### The activities of the LPPC/HLA/Ab complex in vivo and ex vivo

The transgenic mice were vaccinated by IV injection with 6 µg peptides-loaded HLA-A2 and 1.5 µg anti-CD28 mAb on 100 µg LPPC; 6 µg peptides-loaded HLA-A2 on 100 µg LPPC; 100 µg LPPC; 6 µg peptides-loaded HLA-A2; 6 µg peptides-loaded HLA-A2 and 1.5 µg anti-CD28 mAb; or PBS. After two weeks, the mice were individually boosted with the same treatments. Then, one week later, the activity of the immuno-LPPCs was tested. The splenocytes isolated from the mice were cocultured with YML peptides (2 μg/ml) in a 96-well plate at 2.5 × 10^5^ cells per well. The MTT assay was used to estimate the proliferation of cells at 72 h, and ELISA measured the cytokine concentrations of the supernatants.

The specific activities of the LPPC/HLA/Ab complexes (0.6 µg peptides-loaded HLA-A2, and 0.15 µg anti-CD28 or anti-CTLA4 mAb on LPPC) react to splenocytes, the mice prior immunized with YML peptides (30 µg) and Freund's adjuvant*,* were determined based on cell proliferation and cytokine expression.

### Cytotoxicity assay (LPPC/HLA/Ab complex)

Mice were immunized and boosted with immuno-LPPCs as described above. On the 21st day after immunization, splenocytes from treated mice were used as effector cells. In a 96-well plate, TC1/AAD cells as target cells (1 × 10^4^ cells/well) were seeded overnight, and splenocytes were added into the separated wells at E:T ratios of 100:1, 50:1, 25:1, and 12.5:1. After 72 h, the supernatants were removed, and the cells were washed with PBS six times. The MTT assay determined the lysis percentage of target cells, which was calculated by [survival rate of nontreated group (100%)—survival rate of the individual group].

For analysis of immunomodulation efficacy of LPPC/HLA/Ab complexes on splenocyte cytotoxicity, the mice were first immunized and boosted with YML-peptides for three weeks. Then, the mice were treated with LPPC/HLA/Ab complexes once weekly for two weeks. In the 5th week, the MTT assay evaluated the cytotoxicity of the splenocytes against TC1/AAD cells.

### Preparation of lipoplexes with DC membrane proteins and Abs

A previously described method for the isolation and culture of DCs was used [25]. DC membrane proteins (MP) were extracted using a Mem-PER Eukaryotic Membrane Protein Extraction Reagent Kit (Thermo Fisher Scientific Inc., Waltham, MA, USA). For DC MP adsorbed with LPPC, LPPCs (100 µg) were incubated respectively with different amounts of membrane proteins in one tube for 30 min and then centrifuged. The supernatants were removed, and the pellet was resuspended in 100 µl of RPMI solution (Invitrogen).

To form LPPC/MP/Ab complexes, 100 µg of LPPC, different amounts of MP (50, 100, 200 µg), and 1.5 µg anti-CD28 or anti-CTLA4 Abs were co-incubated in one tube for 30 min and centrifuged to remove the supernatants. The pellet was resuspended in 100 µl of RPMI solution for further investigation.

### Immunization with LPPC/MP complex or immunomodulation with LPPC/MP/Ab complex in vivo

Six- to eight-week-old female BALB/c and C57BL/6 mice were purchased from NLAC. The mice were immunized by IV injection with the different treatments, such as 200 µg MP on 100 µg LPPC; 200 µg MP and 1.5 µg anti-CD28 mAb on 100 µg LPPC; 200 µg MP and 1.5 µg anti-CTLA4 mAb on 100 µg LPPC. After two weeks, mice were boosted with the same complex by IV injection. Moreover, under immunomodulation treatments, mice that were immunized and boosted with LPPC/MP complexes were by IV injection of different LPPC/MP/Ab complexes weekly.

### ELISPOT (LPPC/MP/Ab complex)

Mice were immunized and boosted with immuno-LPPC complexes as described above. The investigation of the activity of LPPC/MP complexes was performed by collecting splenocytes from the treated mice 21 days after immunization, and the number of cells expressing IL-4 or IFN-γ was monitored using a U-CyTech BV mouse ELISpot kit (IFN-γ or IL-4; U-CyTech, Yalelaan, The Netherlands) following the manufacturer’s standard protocol. The spots in the wells were visualized and counted using an inverted microscope (Olympus, Shinjuku, Tokyo, Japan).

### Cell proliferation and cytokine ELISA (LPPC/MP/Ab complex)

The mice were immunized and boosted as described above. Splenocytes from treated mice were pulsed with HpHsp60 antigen (1 µg/ml) or EGFP antigens (1 µg/ml) for 72 h, an MTT assay (Sigma‒Aldrich) was estimated cell proliferation, and then the supernatants were analyzed for cytokines (TGF-β, IL-4, and IFN-γ) using ELISA (R&D, Minneapolis, MN, USA).

### Cytotoxicity (LPPC/MP/Ab complex)

As described in our previous study [26], BALB/3T3 cells were transfected with pCJ-*H. pylori* heat shock protein 60 (HpHsp60) or pCJ-HpUreaseB (provided by Dr. Wu, National Taiwan Ocean University, Keelung City, Taiwan, ROC), which expressed HpHsp60 or Urease B, respectively. The transfected BALB/3T3 cells were plated (1 × 10^4^ cells/well in a 96-well plate) and then incubated overnight. Splenocytes obtained from the immunized mice were added to the wells as effector cells at E:T ratios of 100:1, 50:1, 25:1, and 12.5:1. After incubation for 72 h, the cell viabilities were determined by an MTT assay.

B16F10-pLEGFP or B16F10 cells were seeded at 2 × 10^3^ cells/well in a 96-well plate and incubated overnight. Splenocytes were added to the wells as effector cells at an E:T ratio of 100:1. After incubation for 72 h, the cell viabilities were determined by the MTT assay.

### H&E staining

As described previously [21], the organs or tissues of the mice were formalin-fixed, paraffin-embedded and subsequently stained by using histochemical techniques. The tissue slides were observed under a light microscope.

### RNA sequence analysis of mice splenocytes

The mice were immunized and boosted by LPPC/MP complexes with different antibodies, and the RNA collection of procedure was shown in Fig. 9A. Splenocyte RNA was extracted using a PureLinkTM RNA Mini kit (Thermo Fisher Scientific Inc., Waltham, MA). Then, NGS was performed with a NovaSeq 6000 system (Illumina, CA). The raw counts were estimated and normalized as fragments per kilobase per million (FPKM). The gene expression was further selected by *t*-test and ANOVA analysis. All supporting NGS data were available to researchers in the Gene Expression Omnibus (GEO) database, and the accession number was GSE217167.

### Statistical analysis

Data were analyzed using the SAS statistical software package (SAS Institute, Inc., Cary, NC, USA). Two independent trials were compared by a *t-*test, and multiple variables were compared by ANOVA. *P* < 0.05 indicated statistically significant differences. All results are expressed as the mean ± SD.

## Results

### The LPPC/antibody complexes could dually regulate immune responses by the adsorption of immunoregulatory antibodies

**Fig. 1:**
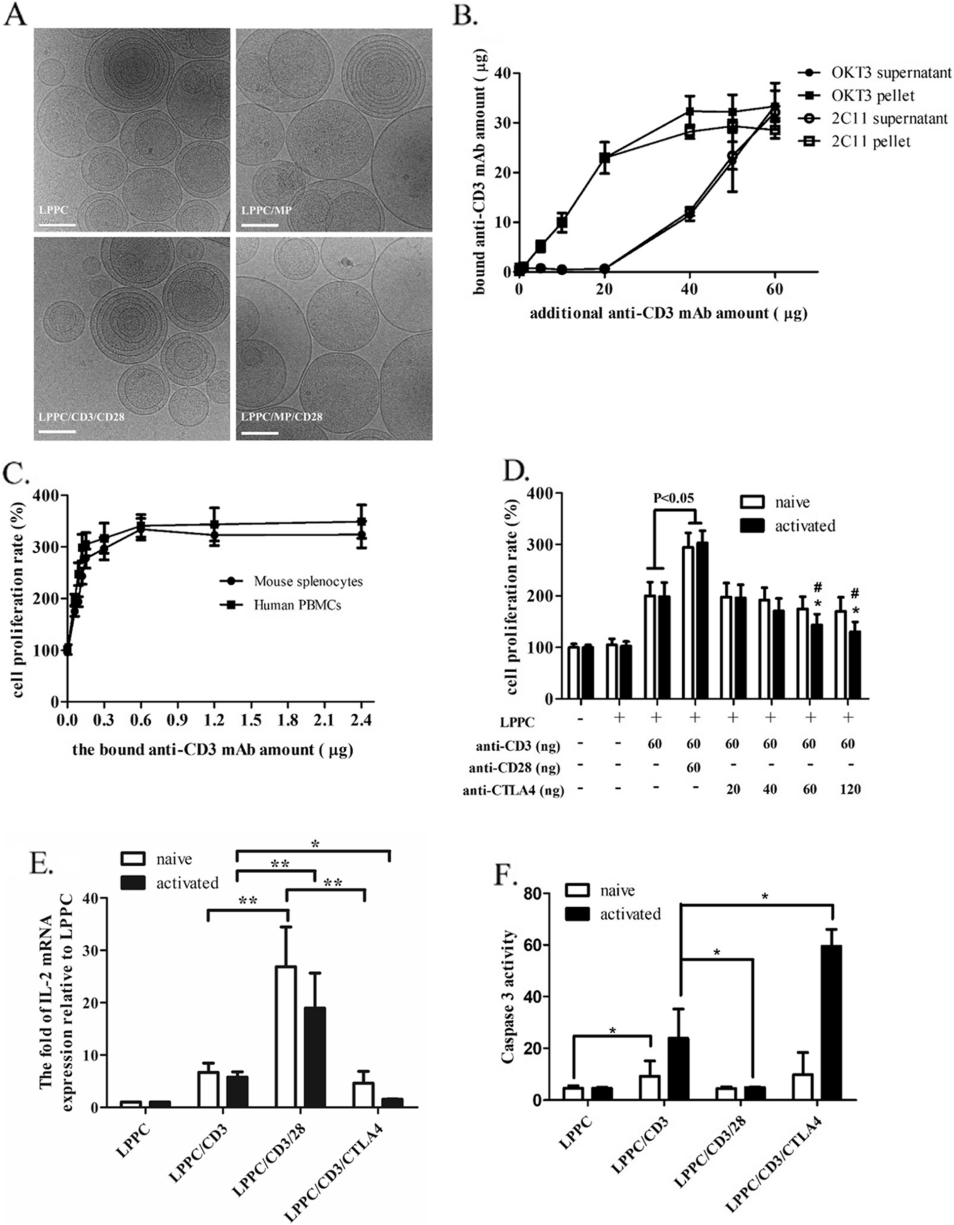
The characteristics of LPPC with bound antibodies. **A** A cryo-TEM instrument was utilized to take images of samples of LPPC complexes (2 mg/ml). Representative images of LPPC complexes were shown (scale bars: 100 nm). **B** The Bradford assay was used to determine the maximum amount of mAb on LPPC. **C** The activities of OKT3 and 2C11 mAbs on LPPC were respectively evaluated the cell proliferation rate by the MTT assay. **D** The cell proliferation rate of naive or activated mice splenocytes under LPPC/Ab complexes treatment was determined by the MTT assay. A significant difference compared to the naive splenocytes group was indicated by * (*P* < 0.05), and comparison to the LPPC/CD3 group is indicated by # (*P* < 0.05). **E** The IL-2 mRNA expressions of splenocytes were determined by real-time quantitative PCR experiments. A significant difference was indicated by * (*P* < 0.05) and ** (*P* < 0.01). **F** The caspase 3 activity as an apoptosis indicator was determined by Ab staining. The fluorescent mean of the cell was shown from the expression of caspase 3

According to previous studies, LPPC could stably adsorb proteins and the bound proteins retain their activities. However, it is still unclear whether LPPC-adsorbed antibodies (Abs) can regulate immunity; therefore, we examined this effect. TEM images showed that LPPC particles were round-shape liposomal particles, and the adsorbed antibodies and proteins increased the diameters from 200 to 300 nm (Fig. 1A). To examine the capacity for Ab adsorption, the anti-human CD3 Ab (OKT3) or anti-mouse CD3 Ab (2C11) was used. Figure 1B showed that the maximal dosages for 10 µg LPPC were approximately 32 µg OKT3 and 28 µg 2C11, respectively. Besides, the activities of the bound anti-CD3 Abs both increased gradually in a dose-dependent manner from 0.15 to 0.6 µg for immune cell proliferation, and the reactions reached a plateau at 0.6 µg bound antibodies on 10 µg LPPC (Fig. 1C). These results revealed that the antibodies bound to LPPC could retain their immunoregulatory activities.

For further regulation of immunity, the immunoregulatory LPPC/antibody complexes were incorporated with anti-CD28 Ab or anti-CTLA4 Ab to achieve positive and negative regulation of the immune response through costimulatory responses, respectively. Figure 1D showed that LPPC with anti-CD3 and anti-CD28 Abs (LPPC/CD3/CD28) could significantly stimulate the proliferation of naive and activated mouse splenocytes more efficiently than the LPPC/CD3 complex. Conversely, LPPC with anti-CD3 and anti-CTLA4 Ab complexes (LPPC/CD3/CTLA4) suppressed the proliferation of activated splenocytes but not naive in a dose-dependent manner (Fig. 1D). In addition, of all the tested groups, LPPC/CD3/CD28 stimulated the highest expression of IL-2 mRNA. In contrast, LPPC/CD3/CTLA4 inhibited IL-2 mRNA expression of activated splenocytes and had no effect on naive splenocytes (Fig. 1E). LPPC/CD3/CD28 treatment also resulted in the lowest cell death for the naive or activated splenocytes, while LPPC/CD3/CTLA4 treatment enhanced the cell death of activated splenocytes but not naive splenocytes (Additional file 2). Figure 1F revealed that LPPC/CD3/CTLA4 promoted cell death by increasing the amount of activated caspase 3 in the activated splenocytes, whereas LPPC/CD3/CD28 suppressed cell death by decreasing the amount of activated caspase 3. Taken together, these findings showed that the immuno-LPPC could positively activate immunity by anti-CD28 Abs or negatively regulate immune responses by anti-CTLA4 Abs.

### The LPPC complex could perform specific immunoregulatory functions by only adsorbing MHC/peptide complex and antibodies

**Fig. 2:**
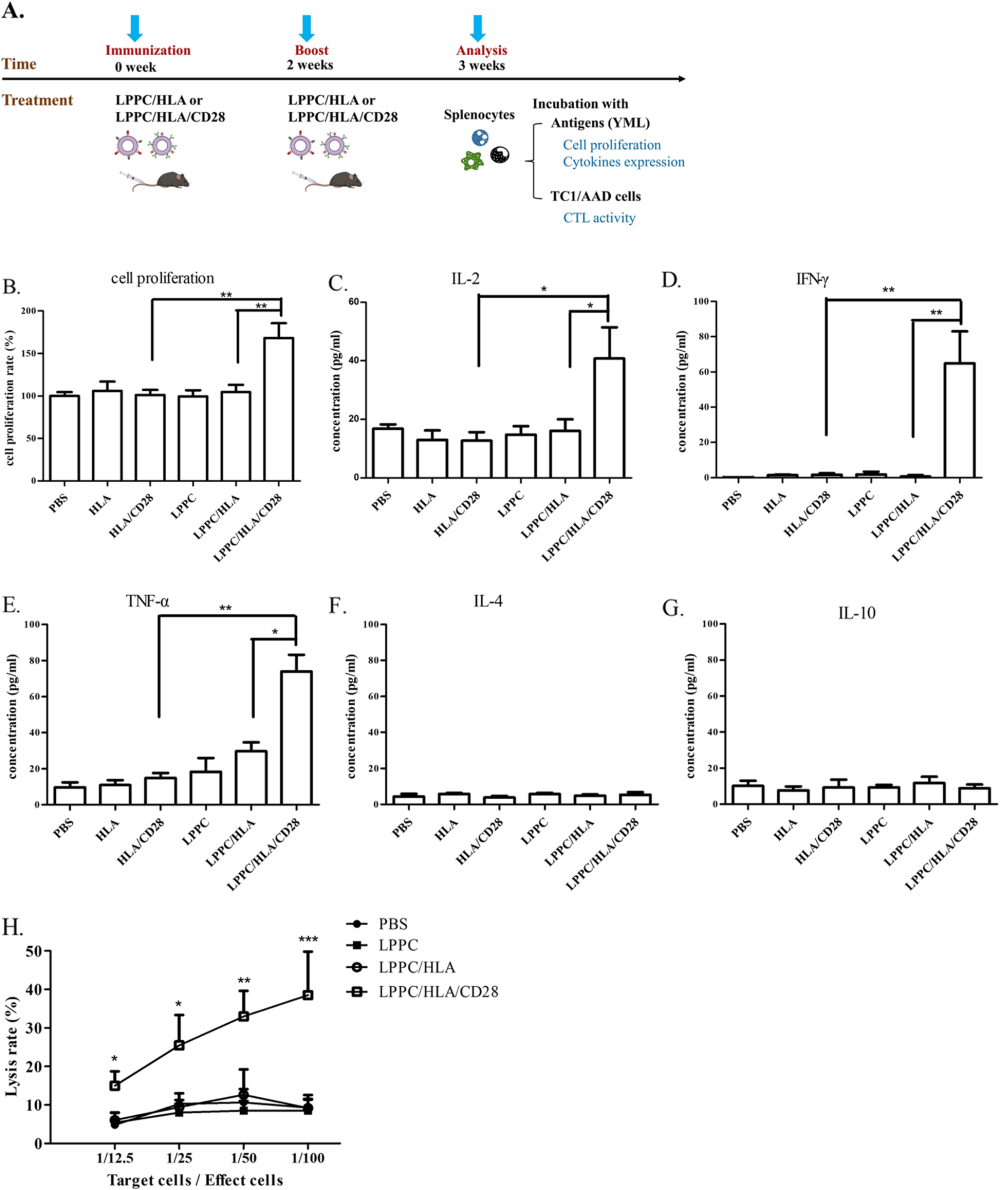
The activities of LPPC with peptides-loaded HLA-A2 molecules and anti-CD28 mAb in vivo. **A** The picture illustrated how to evaluate LPPC/HLA/Ab complexes could stimulate the mice immunity against YML antigens and TC1/AAD cells in vivo. **B-G** The mice with different treatments were determined the immunomodulation efficiency of immuno-LPPC complexes, which included cell proliferation rate and cytokines expressions (IL-2, IFN-γ, TNF-α, IL-4, and IL-10). A significant difference was indicated by * (*P* < 0.05) and ** (*P* < 0.01). Data were expressed as the mean ± SD (*N* = 6). **H** The splenocytes from the mice which were immunized with different treatments incubated with YML-presented TC1/AAD cells. MTT assay was evaluated the cytotoxicity of mice splenocytes. Significant differences were found for the LPPC/HLA/CD28 group compared with the LPPC group (*N* = 6, *: *P* < 0.05, **: *P* < 0.01)

To investigate whether the induction of specific immune response by immuno-LPPC complex, therefore, complexed with recombinant HLA-A2 proteins was to examine its effect. For this, YMLDLQPETT peptides (YML) derived from HPV E7 protein loaded into HLA-A2 molecules were utilized to engage the TCR of T-cell. Complexed with LPPC, Abs, and HLA-A2/YML immunized and boosted the mice, and then the mice splenocytes were determined the responses to YML (Fig. 2A). To trigger active immunity, the results showed that only simultaneously treated with a combination of YML-loaded HLA-A2 molecules and anti-CD28 Abs on LPPC could induce specific T-cell proliferation and Th1 cytokine release, including IL-2, IFN-γ, and TNF-α, but not the Th2 cytokines IL-4 and IL-10 (Fig. 2B-G). Besides, the YML-loaded HLA-A2 molecules could not trigger the immune response in vivo even with anti-CD28 Ab, without LPPC adsorption. Additionally, in the CTL assay, TC1/AAD cells that could present YML peptides in MHC class I were used as the target cells. LPPC/HLA/CD28 complexes stimulated the lytic activity of splenocytes (Fig. 2H).

**Fig. 3:**
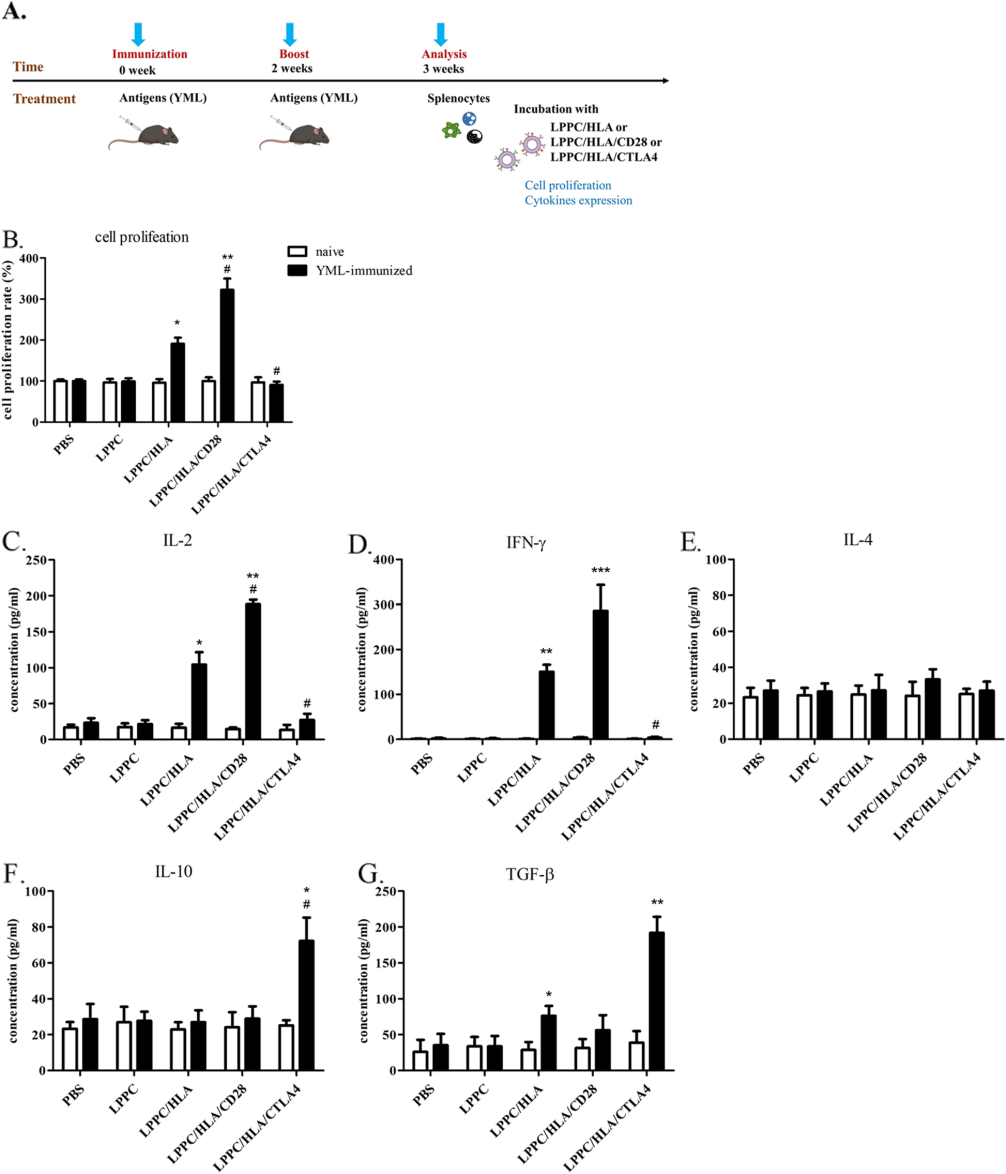
The activities of the immuno-LPPC complex incubated with splenocytes. **A** The picture illustrated how to investigate LPPC/HLA/Ab complexes could react the mice splenocytes that were successfully immunized with YML-peptides. **B-G** LPPC adsorbed peptides-loaded HLA-A2 molecules with or without mAbs co-incubated splenocytes, and the splenocytes had been immunized with YML peptides (solid bar) or not (open bar). The activities of immuno-LPPC were determined by MTT assay for the cell proliferation rate or ELISA for the cytokine expression (IL-2, IFN-γ, IL-4, TGF-β, and IL-10). A significant difference compared with LPPC alone was indicated by * (*P* < 0.05), ** (*P* < 0.01), and *** (*P* < 0.001). A significant difference compared with the LPPC/HLA group was indicated by # (*P* < 0.05). Data were expressed as the mean ± SD (*N* = 6)

Then, YML-immunized splenocytes were further examined ex vivo for the activity of LPPC/HLA/CTLA4 (Fig. 3A). Figure 3 showed that the YML-immunized splenocytes but not naive splenocytes could specifically react with LPPC/HLA, while LPPC/HLA/CD28 complexes caused stronger cell proliferation and Th1 cytokines releases, including IL-2 and INF-γ, than did LPPC/HLA. In contrast, LPPC/HLA/CTLA4 complexes specifically inhibited the cell proliferation and induced the expression of TGF-β and IL-10 in the YML-immunized splenocytes but not the naive splenocytes (Fig. 3B-G).

**Fig. 4:**
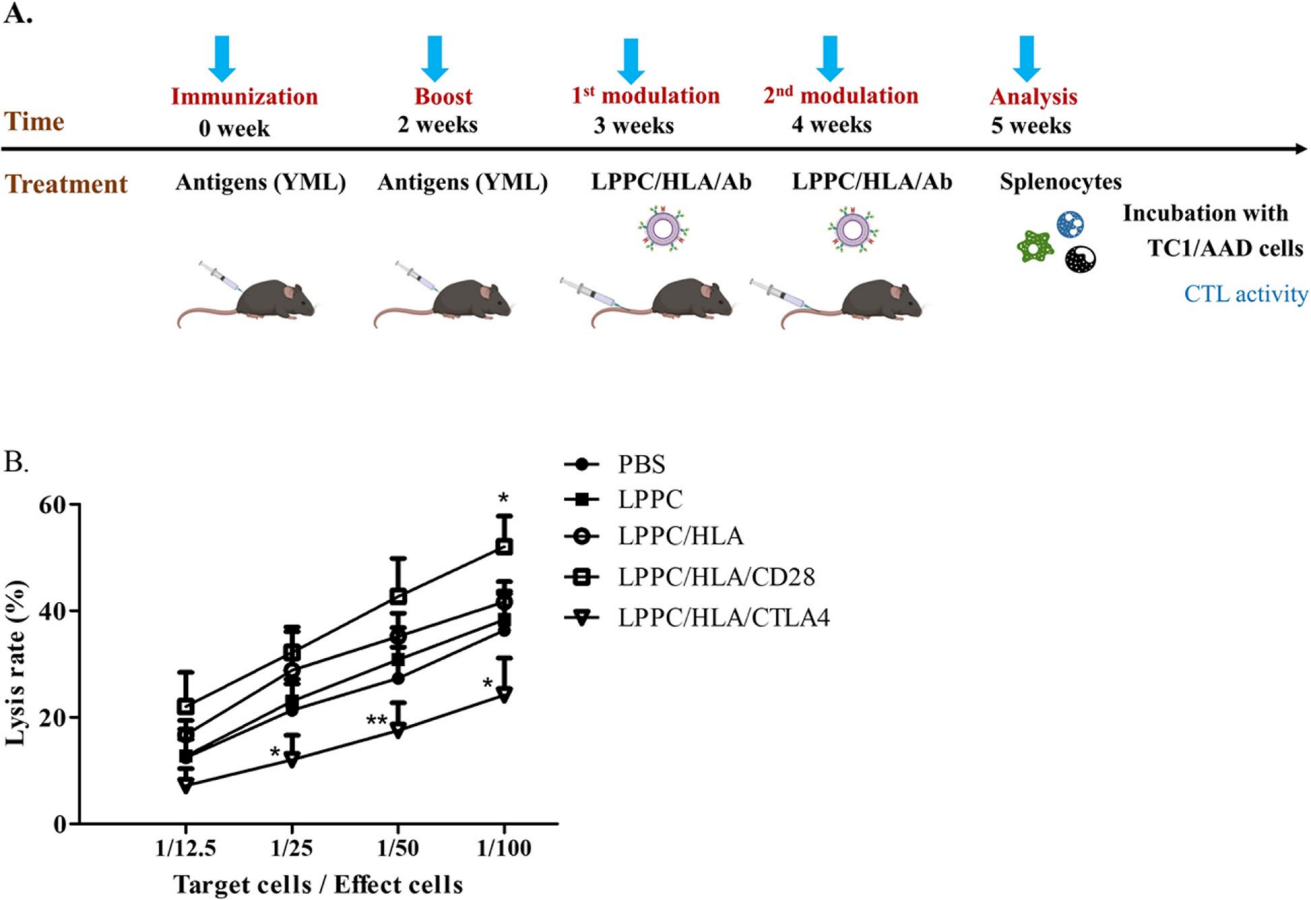
The effects of LPPC in combination with different antibodies on the cytotoxicity of mouse splenocytes. **A** The picture illustrated how to investigate LPPC/HLA/Ab complexes could regulate the cytotoxicity of the mice splenocytes that were successfully immunized with YML-peptides. **B** The cytotoxicity of mouse splenocytes was measured the survival rate of TC1/AAD cells by the MTT assay. Significant differences were compared with the LPPC/HLA group (*N* = 6, *: *P* < 0.05, **: *P* < 0.01)

For in vivo immunoregulation, mice were immunized twice with YML peptides and then regulated twice with LPPC/HLA/Abs (Fig. 4A). One week after regulation, the CTL activities of the splenocytes of treated mice were examined. The results showed that LPPC/HLA/CD28 complexes led to an increase in lysis rate at a 1:100 T/E ratio compared with LPPC/HLA. In contrast, LPPC/HLA/CTLA4 led to a significantly decreased lysis rate compared to that of LPPC/HLA (Fig. 4B). These results revealed that the LPPC immunocomplex could perform immunoregulation only in the existence of two related molecules (MHC/peptide and regulatory Abs) on the LPPC complex.

### The plasma membrane proteins of DC provided an immunoregulatory LPPC complex to induce antigen specificity responses

**Fig. 5:**
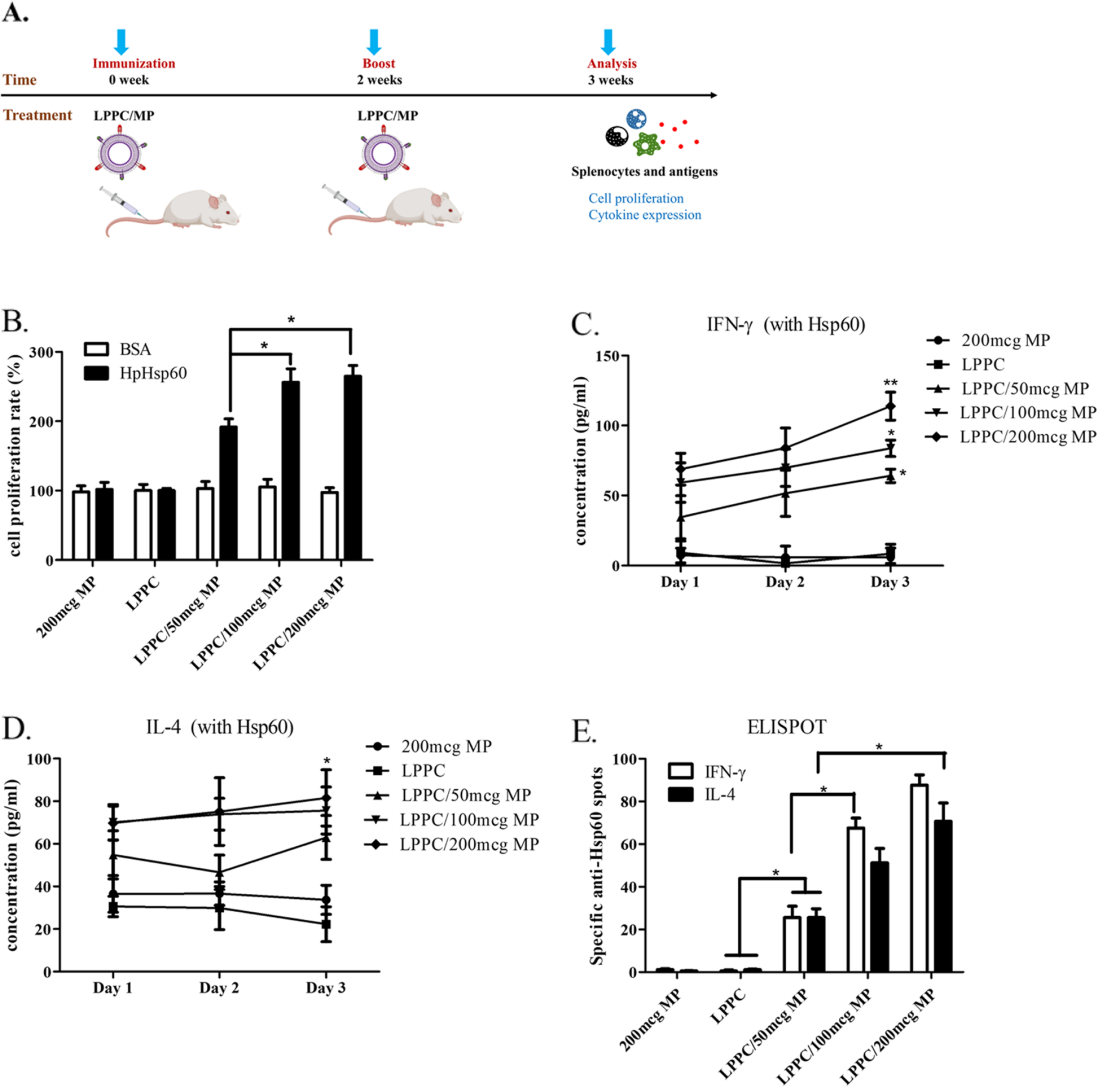
The activities of LPPC/DC MP complexes. **A** The picture illustrated how to prepare the LPPC/MP complexes to treat mice splenocytes in vivo. **B** The cell proliferation rates of splenocytes from different treatments were evaluated by the MTT assay. Microgram was abbreviated as mcg. **C**, **D** The splenocytes from different treatments were pulsed with antigens, such as IFN-γ and IL-4, and cytokine secretion at different times was estimated by ELISA. A significant difference compared to the LPPC group is indicated by * (*P* < 0.05) and ** (*P* < 0.01). Each data indicated the mean ± SD from three independent experiments (*N* = 6). **E** The numbers of IFN-γ or IL-4 expressing T cells responding to HpHSP60 in treated mice were evaluated by an ELISPOT kit. The spots were observed, and the data were calculated and expressed as the mean ± SD (*N* = 6, *: *P* < 0.05)

DCs can efficiently present specific peptides derived from antigens by MHC molecules on the surface to initiate and modulate the activities of specific T-cell immunity with costimulatory molecules. As shown in Fig. 5A, DC membrane protein (MP) extracts on LPPC particles provided the engagement for specific TCR that may facilitate specific T-cell responses including proliferation and cytokine secretion. Immunization with LPPC/MP complexes was used to examine this theory. The results indicated the MP derived from HpHsp60-pulsed DC indeed enabled LPPC to induce cell proliferation in a dose-dependent manner for the specific antigen HpHsp60 but not the nonspecific antigen BSA. In contrast, immunization with free-form MP derived from HpHsp60-pulsed DC did not facilitate the proliferation response to either HpHSP60 or BSA (Fig. 5B).

Further analysis for the polarization of T cells, IFN-γ (Th1), and IL-4 (Th2) release were examined. Only mice immunized with LPPC/MP complexes specifically increased the cytokine release of both IFN-γ and IL-4 for HpHSP60 but not MP alone or LPPC alone (Fig. 5C-D). Additionally, ELISPOT results indicated that immunization with LPPC/MP complexes also increased the numbers of T cells with IFN-γ or IL-4 expression in a dose-dependent manner (Fig. 5E). However, immunization with LPPC/MP complexes did not lead to any immune responses to the nonspecific antigen BSA (Additional file 3). These results demonstrated that LPPC-bound MP could provide more effective immunization to elicit specific T-cell responses in vivo than free-form MP.

### The LPPC/MP complex with the immunoregulatory antibodies modulates immunity in vivo

**Fig. 6:**
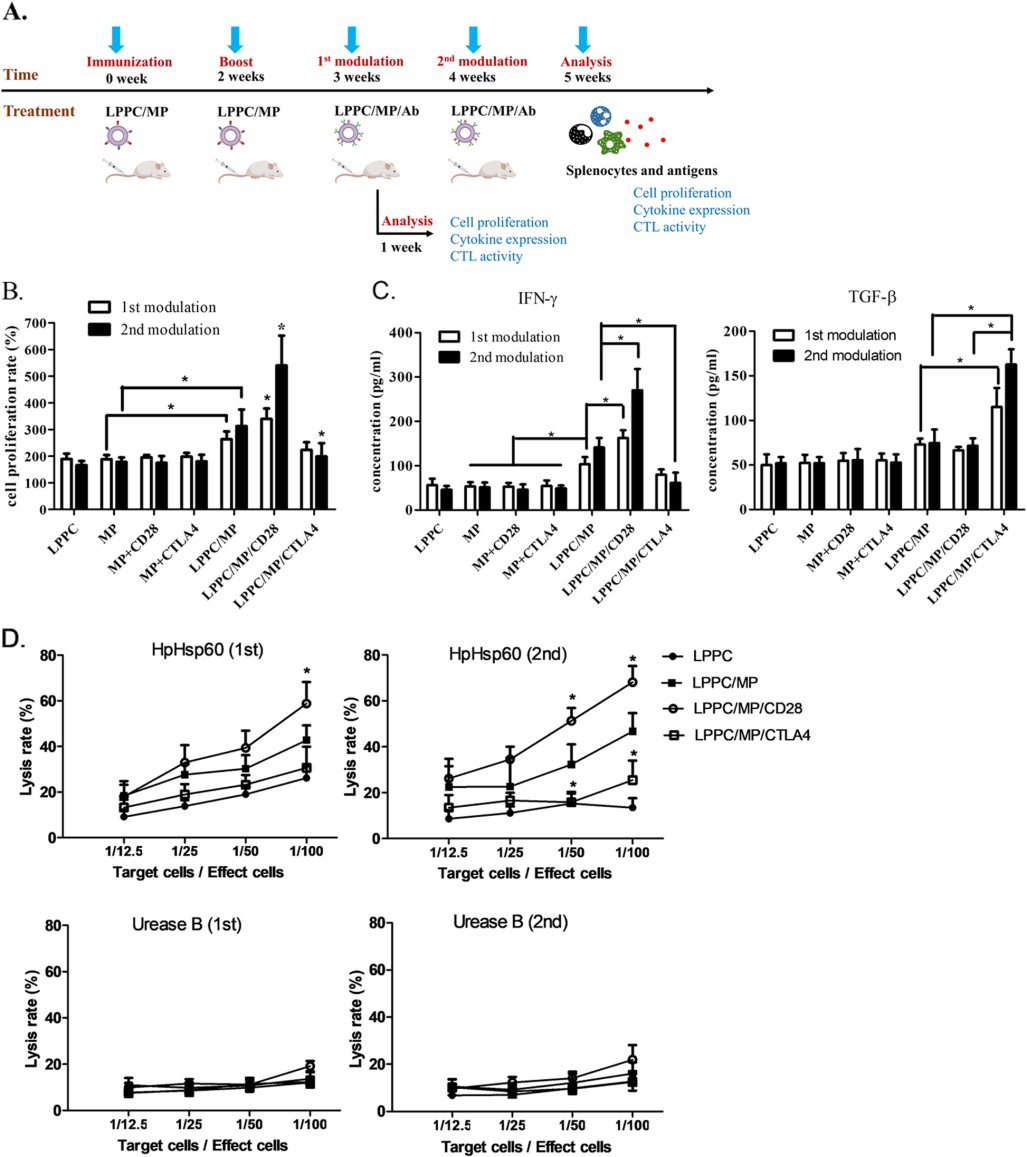
The immunoregulatory effect of LPPC/MP/Ab complexes on mice. **A** The picture illustrated how to evaluate that the LPPC/MP/Ab complexes regulated mice splenocytes, such as cell proliferation, the cytokines expressions, and the cytotoxicity of T cells. **B** The MTT assay was analyzed for the effect of once or twice the modulation of LPPC/MP/Ab complexes on cell proliferation. A significant difference compared to the LPPC/MP group was indicated by * (*P* < 0.05, *N* = 9). **C** The cytokines secretions of the respective supernatants, such as IFN-γ and TGF-β, were evaluated by ELISA (*N* = 9). **D** The cytotoxicity of splenocytes from different mice against pCJ-HpHsp60- or pCJ-HpUreaseB-transfected BALB/3T3 cells were determined. A significant difference compared to the LPPC/MP group was indicated by * (*P* < 0.05, *N* = 9)

Sequentially, the in vivo effects of the regulatory antibodies (anti-CD28 or anti-CTLA4 mAb) with the LPPC/MP complex were determined. Mice were preimmunized with LPPC/MP complexes to establish the anti-HpHSP60 immune response as previously described. Then, the regulatory effects of LPPC/MP incorporated with anti-CD28 (LPPC/MP/CD28) or anti-CTLA4 mAb (LPPC/MP/CTLA4) on these mice were examined. Figure 6B showed that LPPC/MP/CD28 could enhance the proliferation of splenocytes after once or twice treatment compared to LPPC/MP treatment, whereas LPPC/MP/CTLA4 complexes would exhibit their inhibitory function until the second modulation (Fig. 6B). In addition, LPPC/MP/CD28 led to higher IFN-γ but not TGF-β expression, whereas LPPC/MP/CTLA4 stimulated higher TGF-β but not IFN-γ expression (Fig. 6C).

Furthermore, the cytotoxicity assay revealed that LPPC/MP/CD28 increased the activities of HpHSP60-specific CTL against the HpHSP60-expressing cells after the first or second treatment, making it more efficient than LPPC/MP complexes (Fig. 6D). For the suppression of cytotoxicity, LPPC/MP/CTLA4 complexes induced a nonsignificant decrease in specific CTL activity after the first modulation, and a significant reduction in CTL activity after the second modulation compared to LPPC/MP complexes (Fig. 6D, upper panel). However, there was no significant cytotoxicity against the Urease-expressing cells between all treatments (Fig. 6D, down panel). These results revealed that the regulatory antibodies bound on LPPC complex particles would moderate the establishment of specific immunity.

### The immunoregulatory effects of LPPC/MP/Ab complexes on a tumor metastasis model in vivo

**Fig. 7:**
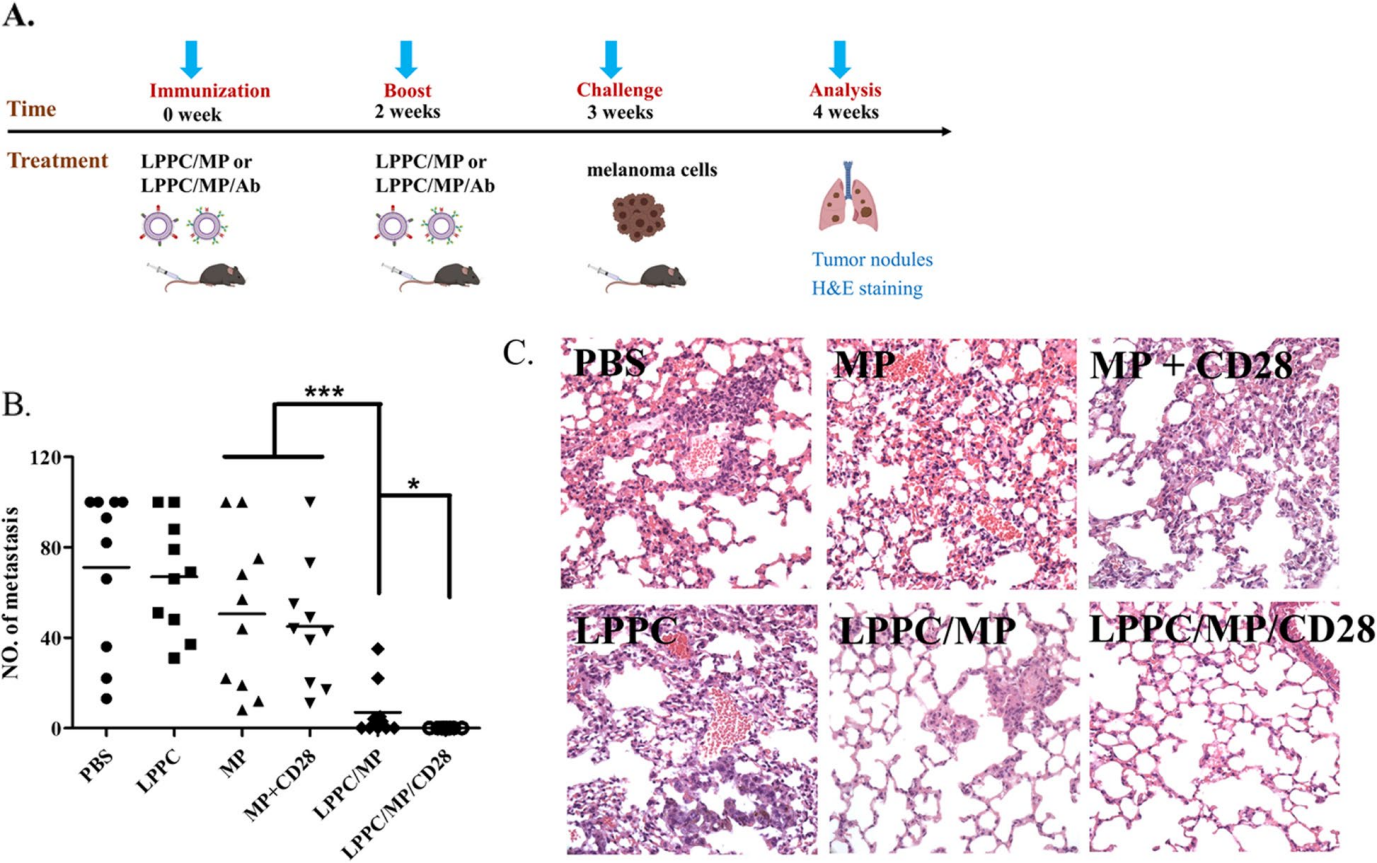
The immune effects of LPPC/MP/Ab complexes on the colony formation of B16F10-pLEGFP cells in mice lungs. **A** The picture illustrated how to prove that LPPC/MP/Ab complexes could regulate mice splenocytes activity against melanoma cells. **B** The number of lung nodules in mice from different treatments were determined. Each data indicated the mean ± SD from three independent experiments (*N* = 10), and a significant difference was indicated by * (*P* < 0.05) and *** (*P* < 0.001). **C** Microscope images of the lungs of mice administrated different treatments were shown

To further evaluate the effects of LPPC/MP/Ab complexes on metastatic tumor cells in vivo, a metastasis model using melanoma cells (B16F10-pLEGFP) was utilized to determine their activities (Fig. 7A). Without LPPC cooperation, immunization with free-form MP or free-form MP plus anti-CD28 antibody did not significantly decrease the numbers of lung nodules, similar to PBS or LPPC treatments (Fig. 7B and Additional file 4A). In contrast, LPPC/MP and LPPC/MP/CD28 complexes both dramatically abolished tumor nodule formation (Fig. 7B). In particular, there were no visible nodules in the lungs of mice after LPPC/MP/CD28 treatments. Except for LPPC/MP/CD28 complexes, histochemical staining clearly revealed that none of the treatments completely impeded the colonization of tumor cells in the lung (Fig. 7C).

**Fig. 8:**
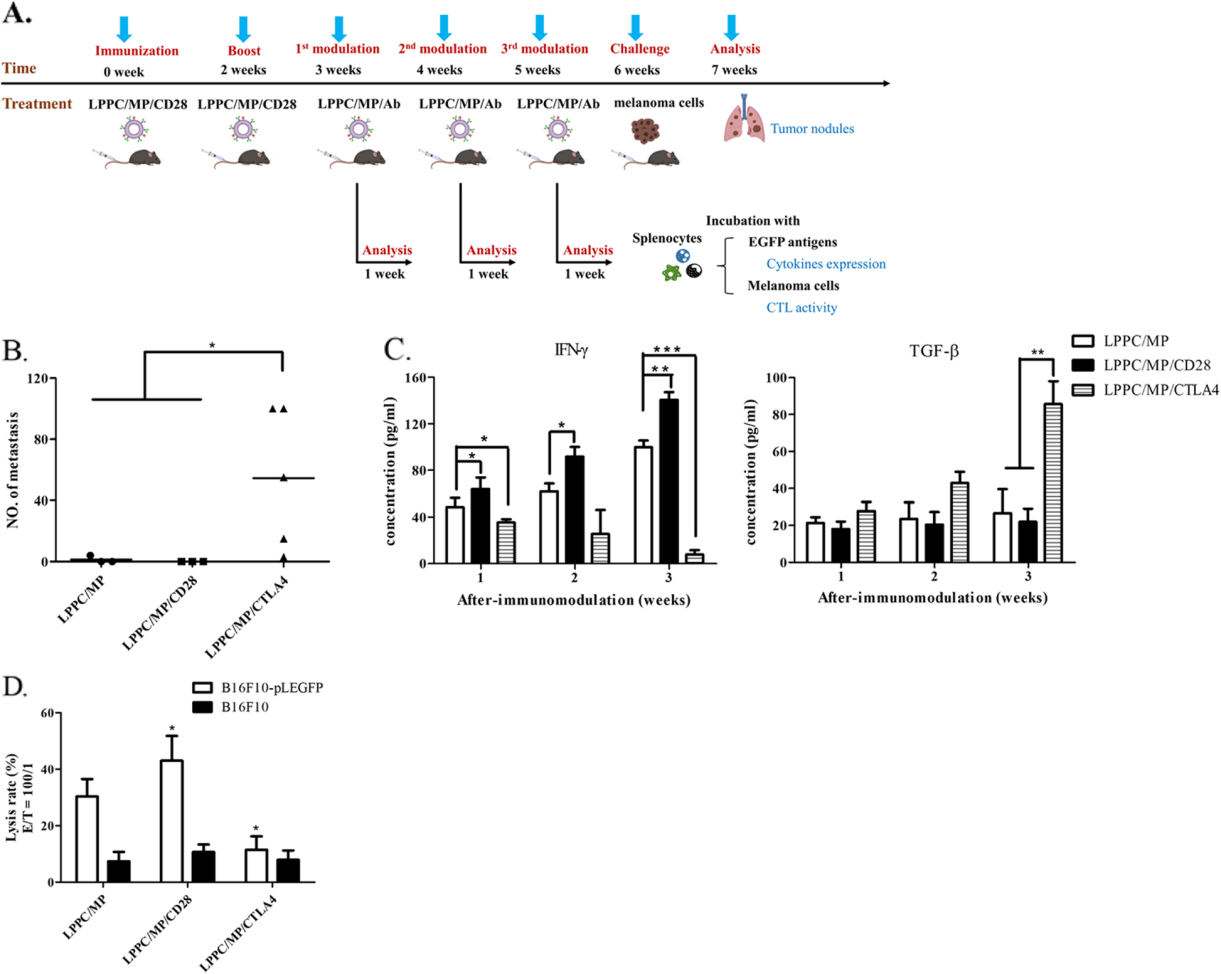
The immunomodulation activities of LPPC/MP complex with different antibodies. **A** The picture illustrated how LPPC/MP/Ab complexes could modulate the anti-tumor immunity of mice splenocytes against melanoma cells in vivo. **B** The number of lung nodules in mice from different treatment groups were determined. A significant difference was indicated by * (*P* < 0.05). **C** After different treatments, splenocytes were harvested from mice at the checkpoint, incubated with EGFP antigen, and the supernatants were analyzed for the expression of IFN-γ and TGF-β (*: *P* < 0.05, **: *P* < 0.01, ***: *P* < 0.001). **D** The cytotoxicity of splenocytes from different mice against B16F10-pLEGFP or B16F10 cells was determined. Statistically significant differences were compared with the LPPC/MP group (*N* = 6, *: *P* < 0.05)

The above results indicated that LPPC/MP/CD28 complexes could efficiently raise host antitumor immunity against metastatic B16F10-pLEGFP cells. Subsequentially, it was determined whether LPPC/MP/CD28-activated immune responses could be erased. Therefore, the mice were previously immunized with LPPC/MP/CD28 complexes twice to establish antitumor immunity (Fig. 8A). One week later, the immunized mice would be modulated with LPPC/MP, LPPC/MP/CD28, or LPPC/MP/CTLA4 weekly for three weeks, and their immune susceptibility to B16F10-pLEGFP cells was examined. At one week after immunomodulation, the mice were inoculated with B16F10-pLEGFP cells by IV injection, and they were sacrificed to monitor tumor colonization in the lung (Fig. 8A). Almost no nodules were found in the lungs of mice treated with LPPC/MP or LPPC/MP/CD28 complexes, consistent with previous results. In contrast, the lung nodules were obvious and severe in the mice treated with LPPC/MP/CTLA4 complexes (Fig. 8B and Additional file 4B). Furthermore, cytokine expression and antitumor cytotoxicity were analyzed. The results showed that the LPPC/MP and LPPC/MP/CD28 complexes increased IFN-γ secretion but had no effects on TGF-β secretion after the 1st immunomodulation, which is consistent with previous results. In contrast, LPPC/MP/CTLA4 treatment not only gradually enhanced TGF-β secretion after the 1st immunomodulation but also decreased IFN-γ secretion (Fig. 8C). Figure 8D further revealed that LPPC/MP/CTLA4 treatment would reduce the CTL to B16F10-pLEGFP cells but not B16F10 cells. These results indicated that the LPPC/MP/Ab complex not only activated specific immunity but also could specifically shut down the established immune response.

### The immunoregulatory effects of LPPC/MP/Ab complexes on gene expression in immune cells in vivo

**Fig. 9:**
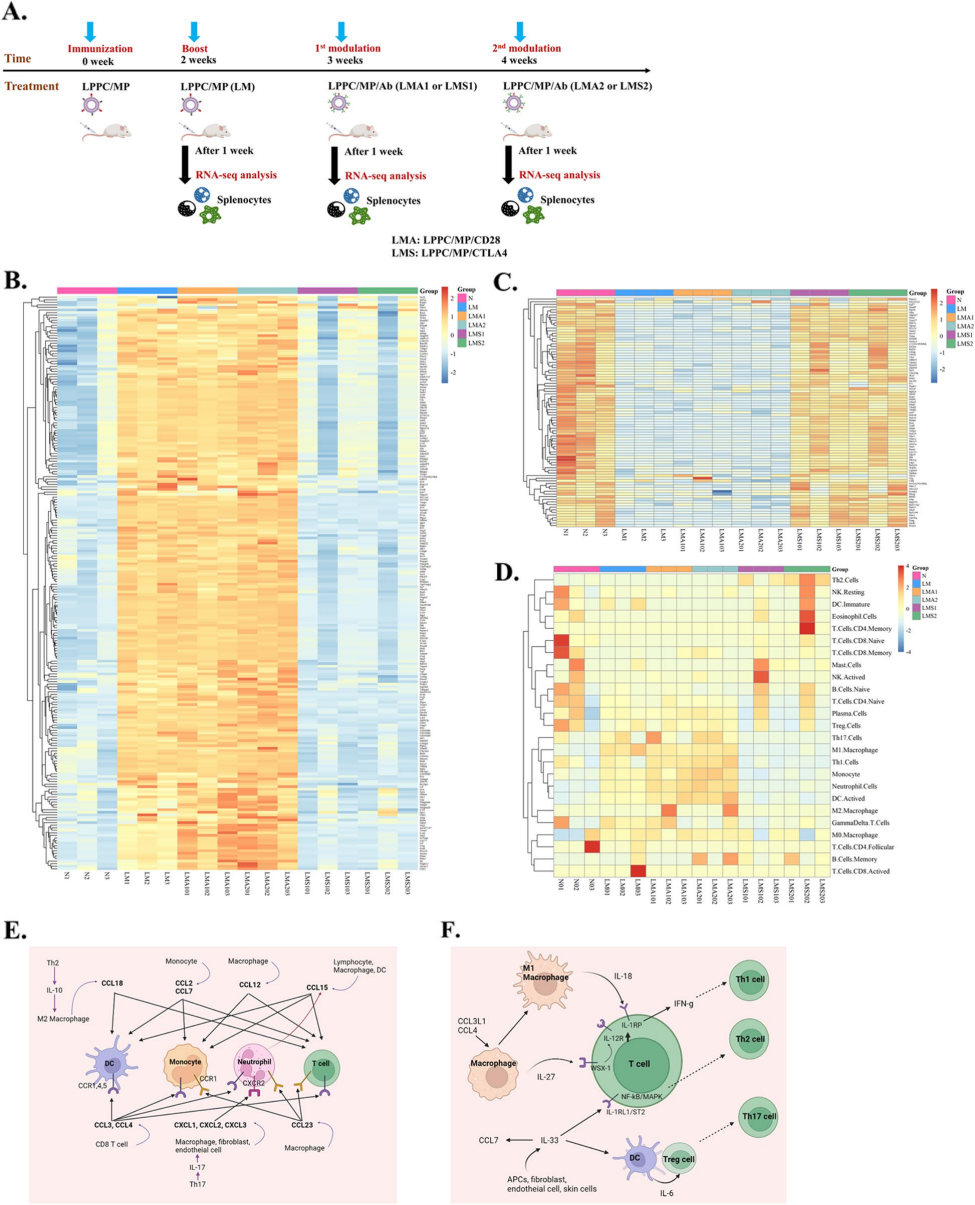
RNA-seq analysis of the immunomodulation activities by LPPC/MP complex with different antibodies. **A**The picture illustrated how to collect the RNA of mice splenocytes after different treatments. **B** and **C**. Heatmap of RNA-seq expression data showing the genes that were differentially regulated under different treatments. N: no-treatment; LM: twice LPPC/MP treatments; LMA1: once LPPC/MP/CD28 treatment; LMA2: twice LPPC/MP/CD28 treatments; LMS1: once LPPC/MP/CTLA4 treatment; LMS2: twice LPPC/MP/CTLA4 treatments. **D** A heatmap of 25 kinds of immune cells under the effects of different treatments, analyzed through the CIBERSORTx website, was shown. The speculated mechanisms and pathways were created using BioRender (https://biorender.com/). The figure showed how LPPC/MP/CD28 complex activated immune responses, according to RNA-seq analysis and related literature. **E** Chemokines and immune cells. **F** Cytokines and immune cells

NGS data revealed that 1712 genes had significant differences in expression between LPPC/MP/Ab and PBS treatments in splenocytes. A flow chart of the RNA-seq data process was shown in Additional file 5. The expression levels and fold changes of these differential expression genes (DEGs) were shown in Additional file 6A. These DEGs were analyzed and annotated with reference to the GeneCards and GO databases and indicated that the major effects of LPPC/MP/Ab were on Immune (18.2%), Signaling (14.3%), Protein metabolism (12.6%), DNA binding (11.3%) and Transport (11.2%) (Additional file 6B). The expression levels of immune-associated DEGs for all samples with different treatments were displayed on the heatmap (Fig. 9B and 9C), and the data showed that compared to PBS treatment, LPPC/MP/CD28 increased the expression levels of 224 genes and decrease the expression levels of 87 genes. In terms of the regulatory effects of the anti-CD28 antibody, LPPC/MP/CD28 significantly increased the expression levels of 34 genes compared to LPPC/MP; the names of these genes were given in Table 2. Interestingly, LPPC/MP/CTLA4 recovered all LPPC/MP-mediated gene expression back to that in the PBS group (Fig. 9B and 9C).

**Table 2:** Significant up- and down-regulation gene expressions between LM and LMA2. LM represented twice LPPC/MP treatments in mice, and LMA2 indicated that twice LPPC/MP/CD28 treatments after LM treatment in mice

	Gene Name	FPKM of LM	FPKM of LMA2	*P*-value (LM v.s. LMA2)
Upregulation	Prss34	4.557	6.883	0.002
	Ms4a3	4.002	6.635	0.005
	Nlrx1	2.279	2.582	0.006
	Mmp8	5.011	8.476	0.010
	Prtn3	16.725	21.497	0.011
	Cd177	10.162	16.802	0.012
	Apbb1ip	74.910	80.799	0.013
	Chil1	3.392	5.876	0.015
	Itgam	93.079	101.966	0.015
	Fcnb	5.854	10.087	0.017
	Mr1	7.138	8.195	0.018
	Gm49368	70.053	76.326	0.020
	Irf7	58.187	72.475	0.020
	Anxa1	176.462	209.383	0.021
	AA467197	1.565	3.047	0.023
	Elane	16.589	23.015	0.023
	Ltf	61.203	99.328	0.023
	Trem3	7.609	10.223	0.024
	Arg1	0.506	0.772	0.026
	Ptges	1.389	1.681	0.027
	Il6	0.572	1.231	0.027
	Cebpb	21.577	23.400	0.030
	Septin5	1.912	2.529	0.030
	Ctsg	8.174	14.154	0.030
	Acod1	32.192	35.813	0.033
	Cxcl2	14.433	18.646	0.033
	Il18	15.711	14.539	0.034
	Gab2	15.537	16.640	0.038
	Pik3ap1	83.432	87.592	0.040
	Cd300e	13.494	14.705	0.042
	Trem1	4.465	7.618	0.047
	Mapk13	0.940	2.014	0.048
	Olfm4	3.476	5.904	0.048
	Cd44	79.985	84.837	0.049
Downregulation	N4bp1	5.237	5.447	0.006
	Erap1	14.454	15.325	0.018
	Det1	2.791	2.309	0.037
	Btbd3	0.362	0.246	0.048
	Smurf2	7.593	6.616	0.049

NGS data also revealed the populations of immune cells through the CIBERSORTx website. A heatmap was shown in Fig. 9D. Compared to LPPC/MP, simultaneously significant increase by LPPC/MP/CD28 and decrease by LPPC/MP/CTLA4 in the populations of immune cells were neutrophils, activated DCs, and monocytes (Additional file 7). Moreover, LPPC/MP/CTLA4 could significantly cause the decreases in the populations of M1 macrophages and γδT cells and the increase in the population of Th2 cells. Additionally, although not statistically significant, LPPC/MP/CD28 seemed to also lead to increases in the Th1 and Th17 cell populations (Additional file 7). Sequentially, KEGG assays were performed to further explore and identify the 64 genes that were increased by the LPPC/MP or LPPC/MP/CD28 but decreased by LPPC/MP/CTLA4 and were involved in chemokine- or cytokine-relative pathways (Fig. 9B). The results showed ten highly enriched pathways (Table 3). According to these NGS data, chemokine and cytokine release might be the reason for the infiltration and activation of T-cell, macrophage, neutrophil and DC populations after LPPC/MP/CD28 treatment. Figure 9E and 9F summarized the effects of chemokines and cytokines on T-cells, macrophages, neutrophils, and DCs.

**Table 3:** The signaling pathways identified by KEGG enrichment analysis. The RNA expression levels of cytokines and chemokines were significantly modulated by LPPC/MP, LPPC/MP/CD28, and LPPC/MP/CTLA4. Ten upregulation pathways and three downregulation pathways were enriched

	ID	Description	GeneRatio	*P*-value
Upregulation	hsa04060	Cytokine-cytokine receptor interaction	24/64	6.64E-19
	hsa04062	Chemokine signaling pathway	13/64	2.06E-09
	hsa04657	IL-17 signaling pathway	11/64	1.17E-10
	hsa04620	Toll-like receptor signaling pathway	10/64	6.21E-09
	hsa04621	NOD-like receptor signaling pathway	10/64	1.40E-06
	hsa04668	TNF signaling pathway	9/64	1.83E-07
	hsa04630	JAK-STAT signaling pathway	8/64	3.49E-05
	hsa04151	PI3K-Akt signaling pathway	8/64	6.12E-03
	hsa04064	NF-kappa B signaling pathway	7/64	1.52E-05
	hsa04659	Th17 cell differentiation	5/64	1.52E-03
Downregulation	hsa04064	NF-kappa B signaling pathway	4/11	7.66E-06
	hsa04620	Toll-like receptor signaling pathway	4/11	7.66E-06
	hsa04010	MAPK signaling pathway	4/11	4.45E-04

## Discussion

This study attempts to address two of the current blockers to tumor immunotherapy for patients; one is to trigger more actively and efficiently specific anti-tumor immunity that involves polymorphic antigen presenting molecules such as MHC, and the heterogenically expressing TAAs in the cancer patient population. By adsorbing anti-CD3 antibody, HLA /peptide, or the MP of autogenic DC with antigen-uptake, these LPPC particles could trigger pan- or specific- T-cell responses to different antigens (HPV E7 peptide, HpHSP60, or EGFP) in different mouse strains with different genetic backgrounds (H-2^d^, human HLA-A2 transgenic or H-2^b^), and they could be dramatically enhanced by anti-CD28 antibody participation. To date, it has been proven that the immuno-microenvironment in tumors dominates the tumor progression or recurrence [27–30], but the findings also facilitate the development of many kinds of immunotherapies for cancer patients [31–33]. In particular, the CAR-technique has evolved several efficient systems and provides dramatic therapeutic benefits to cancer patients [34]. However, the heterogeneity of patients’ TAAs restricts the applicable range for CAR-immunotherapy. TAAs have been identified that are shared among tumor tissues and normal tissues, including mutated gene products, embryonic antigens, and abnormally expressed products [35]. Moreover, the expression profiles of TAAs between individual cancer patients vary and influence therapeutic efficacy [36, 37]. For example, Her-2, an oncogene that is overexpressed in breast or gastric cancer, only happens in about 25% of breast or gastric cancer [8, 9]. Therefore, it is necessary to resolve the heterogeneity of TAAs in patients for immunotherapy. In this platform, the membrane protein extract derived from the autologous DC uptake the TAAs, which could be derived from recombinant protein antigens (Figs. 5–8) or tumor cell extracts; this flexibility may resolve the challenges related to antigen heterogeneity for personalized immunotherapy.

The current secondary blocker is IRAEs, that is, the side effects after immunotherapy; this platform could abolish the activated immunity by switching in the modulating antibodies (anti-CTLA4 antibody) after the activation of antitumor immunity. The experimental evidence showed that LPPC drives the immunoregulatory molecules to eliminate metastatic melanoma cells with high efficacy (Fig. 7). Subsequently, Fig. 8B further showed that such treatment abolished the established antitumor responses to allow cogenetic tumor growth. This active support of the antitumor immune response could be reversed and shut down by adsorbing anti-CTLA4 antibody (Fig. 8). NGS data also indicated that this LPPC-immunoregulatory platform could modulate the immunity by the LPPC particles and their bound regulatory antibodies through the regulation of gene expression (Fig. 9). Importantly, the expression levels of immune-related genes activated by LPPC/MP could be completely reversed back to the levels in the untreated condition, by LPPC/MP/CTLA4 (Fig. 9B and 9C). Therefore, such a therapeutic platform might abolish the severe IRAEs of patients receiving immunotherapy and allow them to further receive other personalized precision therapies. Besides, the immunosuppressive application of this platform may have a potential to develop into a therapy for autoimmune disease; combined with autoantigen(s), this platform should theoretically reverse pathogenic overreactive immunity to normal.

As mentioned above, the immune-modulating mAbs on LPPC could provide their activities to regulate T cells, just as theoretically. Moreover, without LPPC adsorption, the modulating antibodies have no effects on immune responses, suggesting that LPPC is essential in these immunoregulatory responses. Therefore, the LPPC/Ab complex platform may provide more flexible immunoregulation of specific T cells, which are dependent on the activities of the bound antibodies. For example, the CD40/CD40L pathway favors Th1 polarization [38], 4-1BBL with DCs affects the survival and activation of memory CD8 T cells [39], and OX-40L promotes the survival of CD4 T cells [39], all of which can be considered to provide immunomodulation.

Moreover, MP of autologous DC can provide antigen presenting molecules for individual hosts with different genetic backgrounds to trigger specific antitumor responses by properly presenting tumor antigens. At the same time, an interesting event is worthy of focus: In addition to MHC molecules, LPPC/MP complexes could also provide costimulatory B7 molecules to activate host immunity against metastatic tumor cells by engaging CD28 on T-cell (Fig. 7). Furthermore, LPPC/MP/CD28 complexes more dramatically eliminated metastatic tumor cells in the lung (Fig. 7) and stimulated more IFN-γ release than LPPC/MP complexes (Fig. 6). Why can LPPC/MP/CD28 complexes contribute more efficiently to activating T cells than the original ligand B7 molecules of CD28 on MP of DC? This may be due to the following mechanisms: First, the higher affinity of the anti-CD28 antibody for the CD28 molecule may provide better signaling than B7. Second, unlike B7 binding to competitive molecules such as CTLA-4 on T cells, the anti-CD28 antibody does not trigger the inactive response of activated T-cells. Third, the anti-CD28 antibody and B7 can synergistically activate T-cells, because they bind to different domains on CD28. Therefore, using the regulatory antibody in this platform is superior to using the original ligands of those costimulatory receptors.

In addition, the results showed that LPPC is essential in this platform. DC MP containing many MHC/peptide complexes and costimulatory molecules can potentially engage the TCR and activate T-cells [3, 40]. However, LPPC-bound MP could significantly induce immune responses in this study, but MP alone could not (Fig. 5). Again, similar results showed that LPPC/HLA/CD28 could activate T-cell proliferation and IL-2 expression, but HLA/peptide complex and anti-CD28 mAb could not (Fig. 2). Several possibilities have been proposed to explain the mechanism of this phenomenon. Dr. Steve Roffler and colleagues have demonstrated that the TCR complex needs physical forces to initiate signaling for the activation of T cells [41, 42]. DC MP or HLA/peptide complex fused with LPPC complexes could provide pull or drag forces (physical forces) between T cells and LPPC/MP or LPPC/HLA/peptide complexes. Moreover, the DC MP or HLA/peptide complex alone would be diluted in blood to result in a lower concentration of ligand than was present on LPPC complexes [43]. Furthermore, the optimal activation of a T-cell requires two distinct signals derived from the TCR and costimulatory receptors or different immunoregulatory molecules [3]. For example, B7 molecules on APCs can engage with CD28 molecules of T cells to synergistically activate T-cell and induce cell proliferation and IL-2 mRNA expression with TCR [22, 44]. In contrast, TCR-alone (without costimulatory signaling) will cause T cell anergy or apoptosis [45]. Consistently, Fig. 1 showed that the combination of anti-CD3 and anti-CD28 mAbs on LPPC had better effects on cell proliferation and IL-2 mRNA expression, and LPPC with anti-CD3 mAb alone led to apoptosis (higher sub-G1) (Additional file 2). Thus, the adsorption of such effective molecules on LPPC could simultaneously satisfy this requirement.

Our NGS results also indicated that LPPC/MP or LPPC/MP/CD28 could increase the populations of T-lymphocyte, monocyte, M1 macrophage, neutrophil, and activated DC, whereas LPPC/MP/CTLA4 decreased these cell populations. In addition, LPPC/MP/CTLA4 also increased the Th2 cell population and decreased γδT cell population (Additional file 7). According to the published literature and GeneCards information, we proposed that our platform briefly regulated immunity by acting on different T-cells. CD8 + T cells would release MIP-1 αβ to chemoattract T-lymphocytes, monocytes, neutrophils, and activated DCs through CCR-1, 4, 5 [46, 47]. LPPC/MP-activated Th17 cells would trigger fibroblast cells or macrophages to release GRO αβγ, which is a chemoattractant for neutrophils [48]. Furthermore, LPPC/MP/Ab might mediate Th2 cells to express IL-10, which could convert macrophages into M2 cells to express CCL18 and chemoattract DC or T-cell to the target site. Besides, these chemoattracted immune cells might express CCL2, CCL7, CCL12, CCL15 and CCL23 to strengthen immune cell infiltration into the target site (Fig. 9E). Moreover, Fig. 9F showed the MIP-1 αβ-activated macrophages might express IL-27 to increase the expression of IL-12 receptor (IL-12R) through WSX-1/CD130c and convert T cells to Th1 cells [49]. Additionally, these MIP-1 αβ-activated macrophages might express IL-18, which is an IFN-γ inducing factor in T cells, and could trigger T-cell differentiation into Th1 cell [50]. Cells in the microenvironment, including macrophages and fibroblast cells, could express IL-33 to enhance the differentiation of T cells into Th2 cells through IL-1RL1/ST2/NF-ĸB pathway and Treg cells into Th17 cells by acting on DC [51, 52]. Besides, IL-33 could be an inducer of CCL7 [53]. According to these references, we proposed that LPPC/MP/CD28 suppressed the B16F10 tumor growth by mediating chemokine-cytokine interaction.

## Conclusions

This study showed that LPPC combined DC MP and mAbs could be a good tool for regulating an individual’s immune responses by turning specific immune responses on or off. In addition, our previous results showed LPPC complexes could be safe in vivo because no obvious side effects were found in vivo treatment by histopathologic assay [15, 17, 21]. Moreover, to resolve the diversity and variation of MHC molecules and antigens in human population, LPPC/MP/Ab complexes showed remarkable potential and technological advances in human diseases such as cancers or autoimmune diseases for personalized precision immunotherapy. Thus, this platform of immunomodulator is worth developing in the future as a promising therapeutic tool for human diseases because of its flexibility and regulability.

## Supplementary Information

**Additional file 1.** Construction of LPPC/MP andLPPC/MP/Ab complexes.The picture illustrated the formulation of LPPC complexes with different immunofunctionalproteins and their specific aims.

**Additional file 2.** The effects of LPPC/Abcomplexes on the cell cycle. The cell cycle profiles ofnaive (A) or activated (B) splenocytes under LPPC/Ab complexestreatments were performed by PI staining. A significant difference compared tothe LPPC/CD3 group was indicated by * (*P*<0.05).

**Additional file 3.** The activities of LPPC/MP complexes. The splenocytes from different treatments were pulsed with BSA antigens, and the cytokines secretion were estimated at different times by ELISA, such as IFN-γ (A) and IL-4 (B). Each data indicated the mean ± SD from three independent experiments (*N*=6).

**Additional file 4.** The lung photos of mice under different treatments. (A)The representative photos of the lungs from differenttreatments of Fig. 7B were selected. (B) The representative photos ofthe lungs from different treatment groups of Fig. 8B were selected.

**Additional file 5.** Flow chart of theprocess for RNA-seq analysis. The flow chart described how RNA data wereprocessed, the criteria for every stage, and the number of genes. The number ofupregulation genes was shown in red, while that of downregulation genes wasshown in blue.

**Additional file 6.** RNA-seq analysis of theimmunomodulation activities by LPPC/MP complex with different antibodies. (A) Volcano plot for gene expression and the FC values of genes. (B) Pie chart demonstrating the proportionof mouse RNA-seq reads assigned to annotated genomic functions.

**Additional file 7.** RNA-seq analysis of the immunomodulation activitiesby LPPC/MP complex with different antibodies. The RNA expression levels of immunecells under different treatments were determined by *t*-tests and ANOVA,and the results were shownas boxplots. The description of group names was the same as in Fig. 9.

## Data Availability

The datasets used and/or analyzed during the current study are available from the corresponding author upon reasonable request.

## Abbreviations

DC: Dendritic cell
MP: Membrane protein
APC: Antigen presenting cell
CAR-T: Chimeric antigen receptor T cell
LPPC: Lipo-PEG-PEI-complex
TAAs: Tumor associated antigens
IRAEs: Immune-related adverse events
CTL: Cytotoxic T lymphocyte
NGS: Next-generation sequencing
DEGs: Differential expression genes
ICI: Immune checkpoint inhibitors
