# Supplementary material for: A flexible liposomal polymer complex as a platform of specific and regulable immune regulation for individual cancer immunotherapy
Source: J Exp Clin Cancer Res. 2023 Jan 23;42:29. doi: 10.1186/s13046-023-02601-8 (PMC9869520; doi:10.1186/s13046-023-02601-8)
Supplement: Supplementary file 1 — Additional file 1. Construction of LPPC/MP andLPPC/MP/Ab complexes.The picture illustrated the formulation of LPPC complexes with different immunofunctionalproteins and their specific aims. [file 13046_2023_2601_MOESM1_ESM.docx]

**Additional file**

**
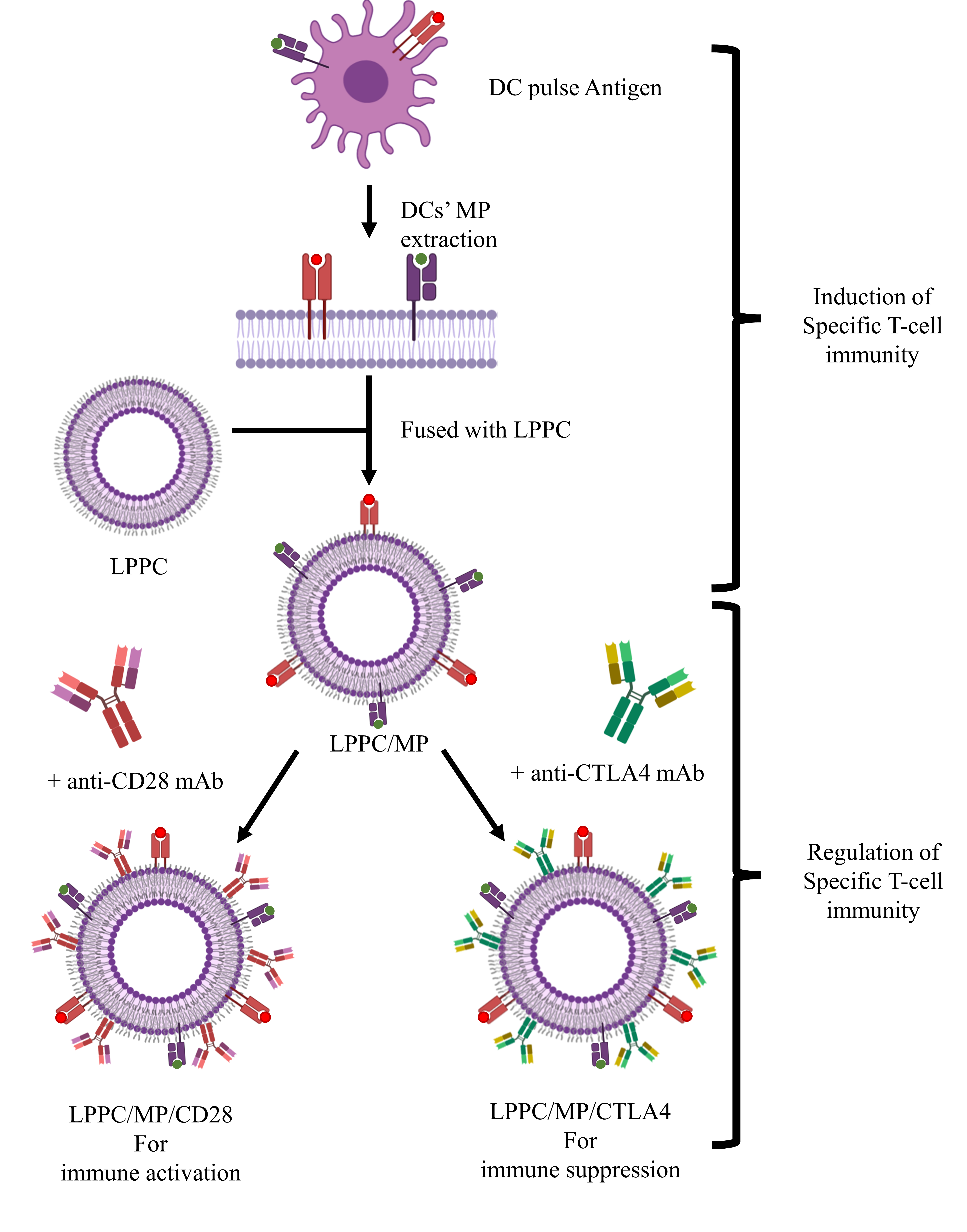
**

**Additional file 1. Construction of LPPC/MP and LPPC/MP/Ab complexes.**

The picture illustrated the formulation of LPPC complexes with different immunofunctional proteins and their specific aims.
