# Supplementary material for: A flexible liposomal polymer complex as a platform of specific and regulable immune regulation for individual cancer immunotherapy
Source: J Exp Clin Cancer Res. 2023 Jan 23;42:29. doi: 10.1186/s13046-023-02601-8 (PMC9869520; doi:10.1186/s13046-023-02601-8)
Supplement: Supplementary file 2 — Additional file 2. The effects of LPPC/Abcomplexes on the cell cycle. The cell cycle profiles ofnaive (A) or activated (B) splenocytes under LPPC/Ab complexestreatments were performed by PI staining. A significant difference compared tothe LPPC/CD3 group was indicated by * (P<0.05). [file 13046_2023_2601_MOESM2_ESM.docx]

**
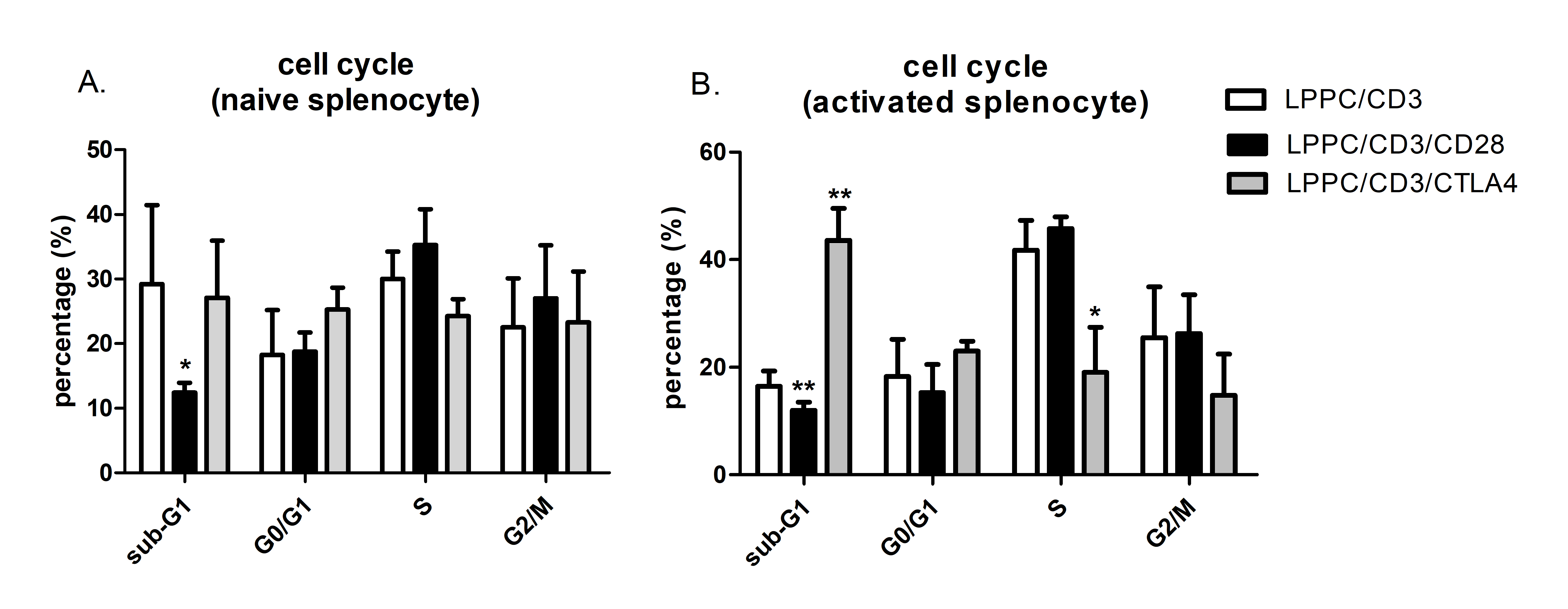
**

**Additional file 2. The effects of LPPC/Ab complexes on the cell cycle.**

The cell cycle profiles of naive **(A)** or activated **(B)** splenocytes under LPPC/Ab complexes treatments were performed by PI staining. A significant difference compared to the LPPC/CD3 group was indicated by * (P<0.05).
