# Supplementary material for: A flexible liposomal polymer complex as a platform of specific and regulable immune regulation for individual cancer immunotherapy
Source: J Exp Clin Cancer Res. 2023 Jan 23;42:29. doi: 10.1186/s13046-023-02601-8 (PMC9869520; doi:10.1186/s13046-023-02601-8)
Supplement: Supplementary file 3 — Additional file 3. The activities of LPPC/MP complexes. The splenocytes from different treatments were pulsed with BSA antigens, and the cytokines secretion were estimated at different times by ELISA, such as IFN-γ (A) and IL-4 (B). Each data indicated the mean ± SD from three independent experiments (N=6). [file 13046_2023_2601_MOESM3_ESM.docx]

**
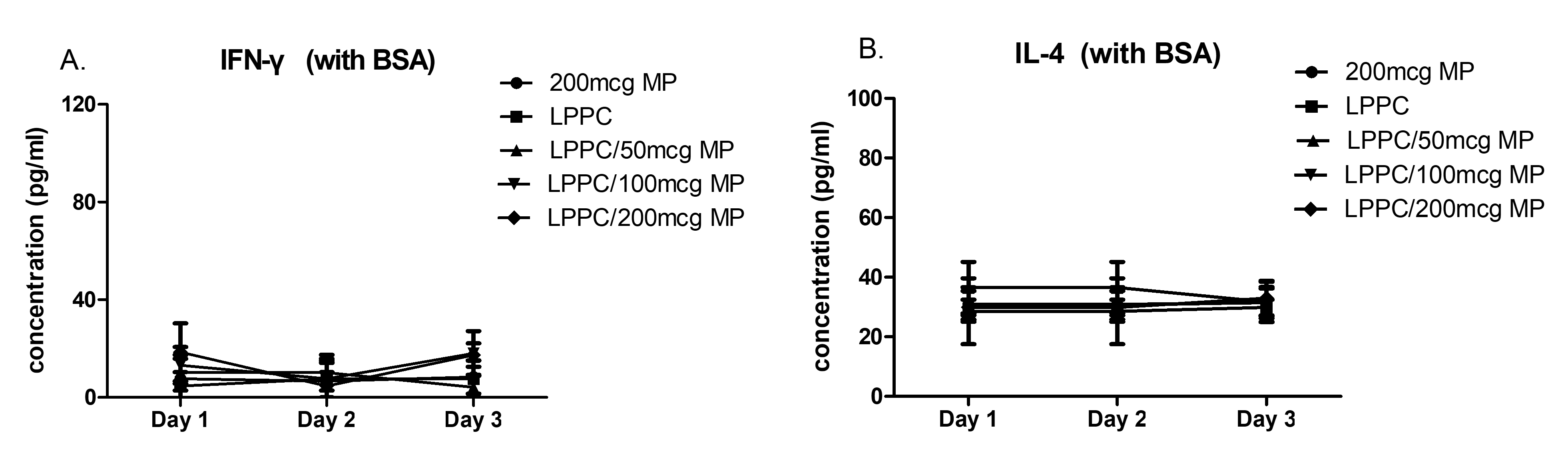
**

**Additional file 3. The activities of LPPC/MP complexes.**

The splenocytes from different treatments were pulsed with BSA antigens, and the cytokines secretion were estimated at different times by ELISA, such as IFN-γ **(A)** and IL-4 **(B)**. Each data indicated the mean ± SD from three independent experiments (N=6).
