# Supplementary material for: A flexible liposomal polymer complex as a platform of specific and regulable immune regulation for individual cancer immunotherapy
Source: J Exp Clin Cancer Res. 2023 Jan 23;42:29. doi: 10.1186/s13046-023-02601-8 (PMC9869520; doi:10.1186/s13046-023-02601-8)
Supplement: Supplementary file 4 — Additional file 4. The lung photos of mice under different treatments. (A)The representative photos of the lungs from differenttreatments of Fig. 7B were selected. (B) The representative photos ofthe lungs from different treatment groups of Fig. 8B were selected. [file 13046_2023_2601_MOESM4_ESM.docx]

**
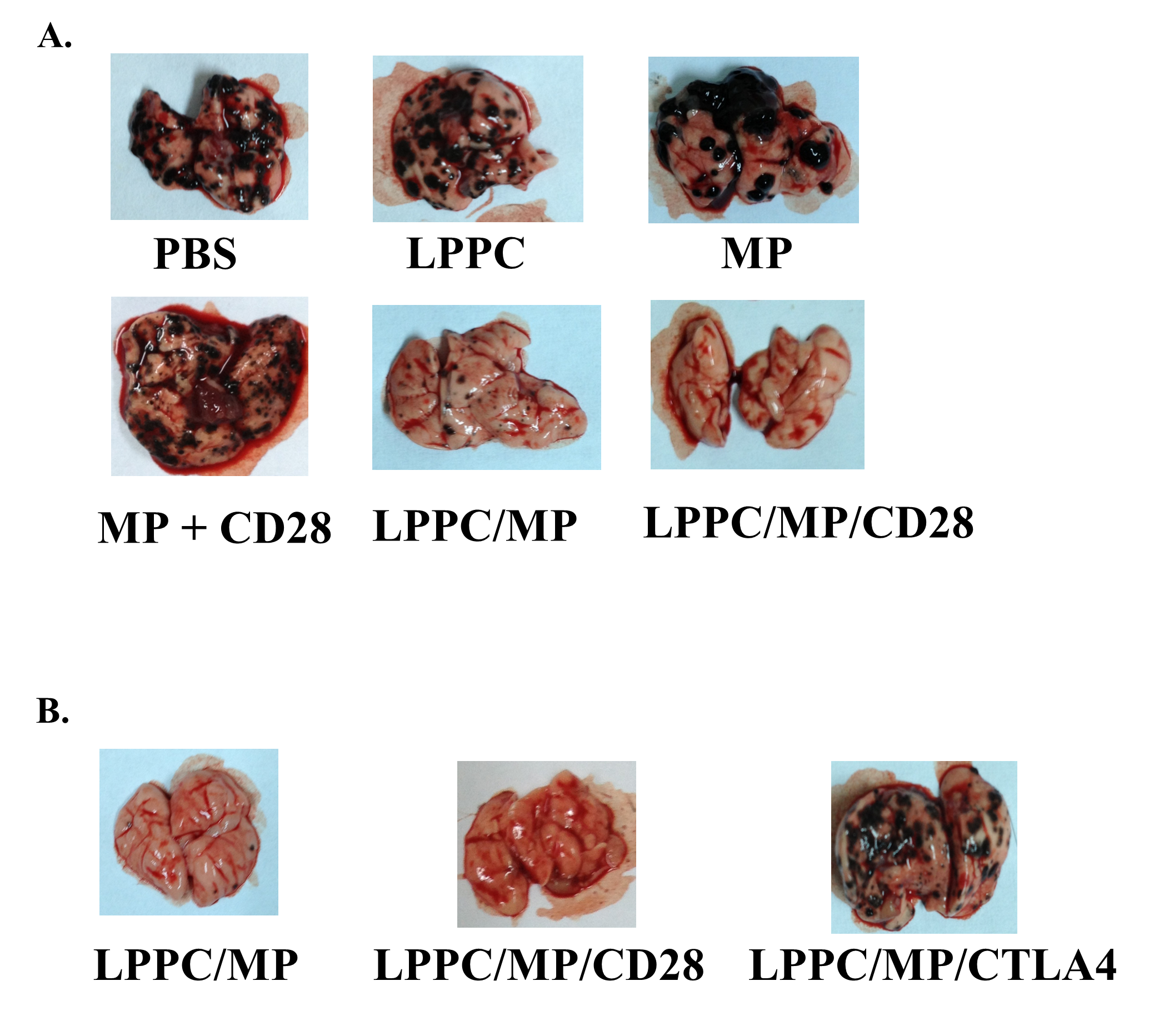
**

**Additional file 4. The lung photos of mice under different treatments.**

**(A)**The representative photos of the lungs from different treatments of Fig. 7B were selected. **(B)** The representative photos of the lungs from different treatment groups of Fig. 8B were selected.
