# Supplementary material for: A flexible liposomal polymer complex as a platform of specific and regulable immune regulation for individual cancer immunotherapy
Source: J Exp Clin Cancer Res. 2023 Jan 23;42:29. doi: 10.1186/s13046-023-02601-8 (PMC9869520; doi:10.1186/s13046-023-02601-8)
Supplement: Supplementary file 5 — Additional file 5. Flow chart of theprocess for RNA-seq analysis. The flow chart described how RNA data wereprocessed, the criteria for every stage, and the number of genes. The number ofupregulation genes was shown in red, while that of downregulation genes wasshown in blue. [file 13046_2023_2601_MOESM5_ESM.docx]

**
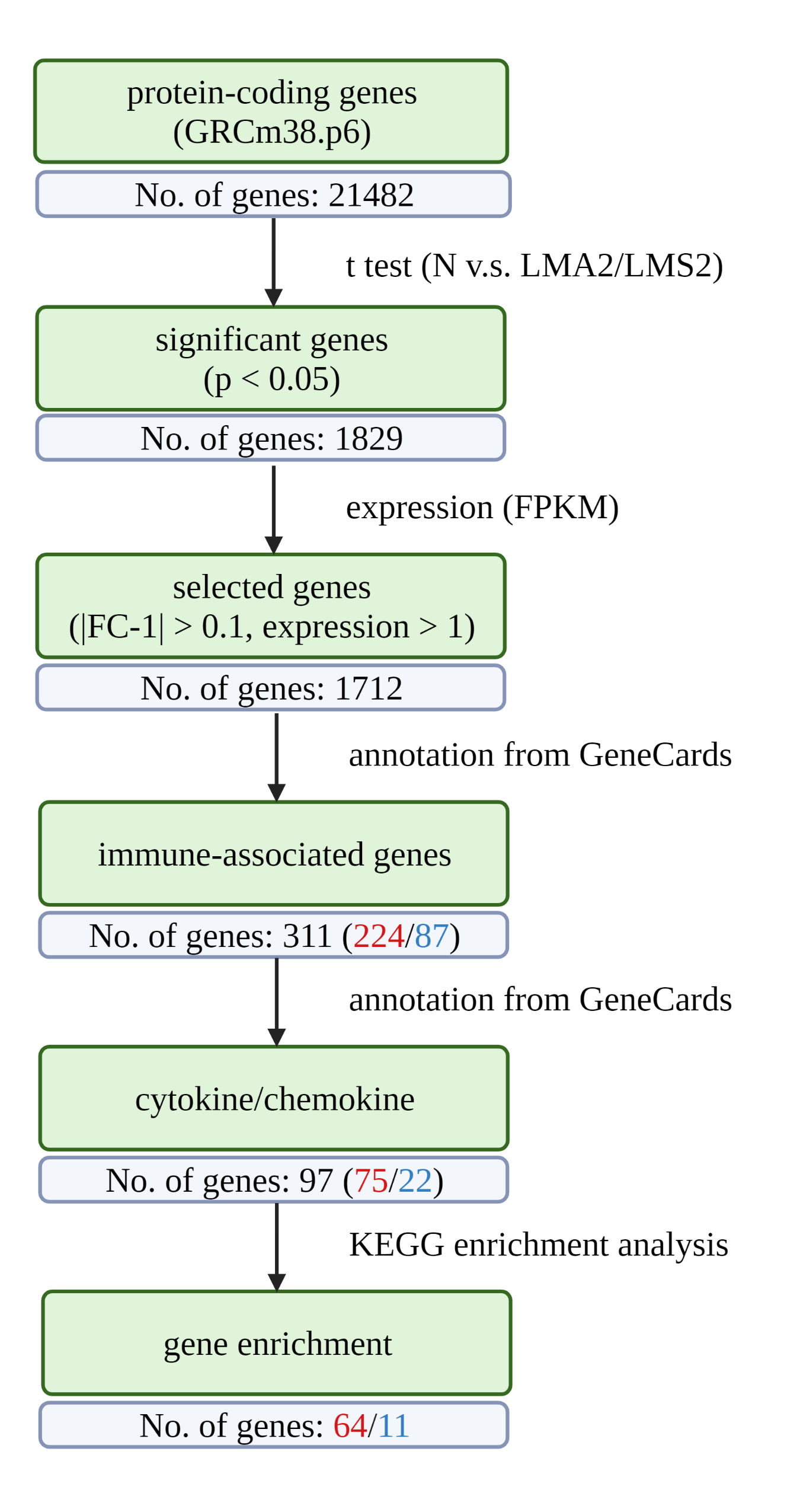
**

**Additional file 5. Flow chart of the process for RNA-seq analysis.**

The flow chart described how RNA data were processed, the criteria for every stage, and the number of genes. The number of upregulation genes was shown in red, while that of downregulation genes was shown in blue.
