# Supplementary material for: A flexible liposomal polymer complex as a platform of specific and regulable immune regulation for individual cancer immunotherapy
Source: J Exp Clin Cancer Res. 2023 Jan 23;42:29. doi: 10.1186/s13046-023-02601-8 (PMC9869520; doi:10.1186/s13046-023-02601-8)
Supplement: Supplementary file 6 — Additional file 6. RNA-seq analysis of theimmunomodulation activities by LPPC/MP complex with different antibodies. (A) Volcano plot for gene expression and the FC values of genes. (B) Pie chart demonstrating the proportionof mouse RNA-seq reads assigned to annotated genomic functions. [file 13046_2023_2601_MOESM6_ESM.docx]

**
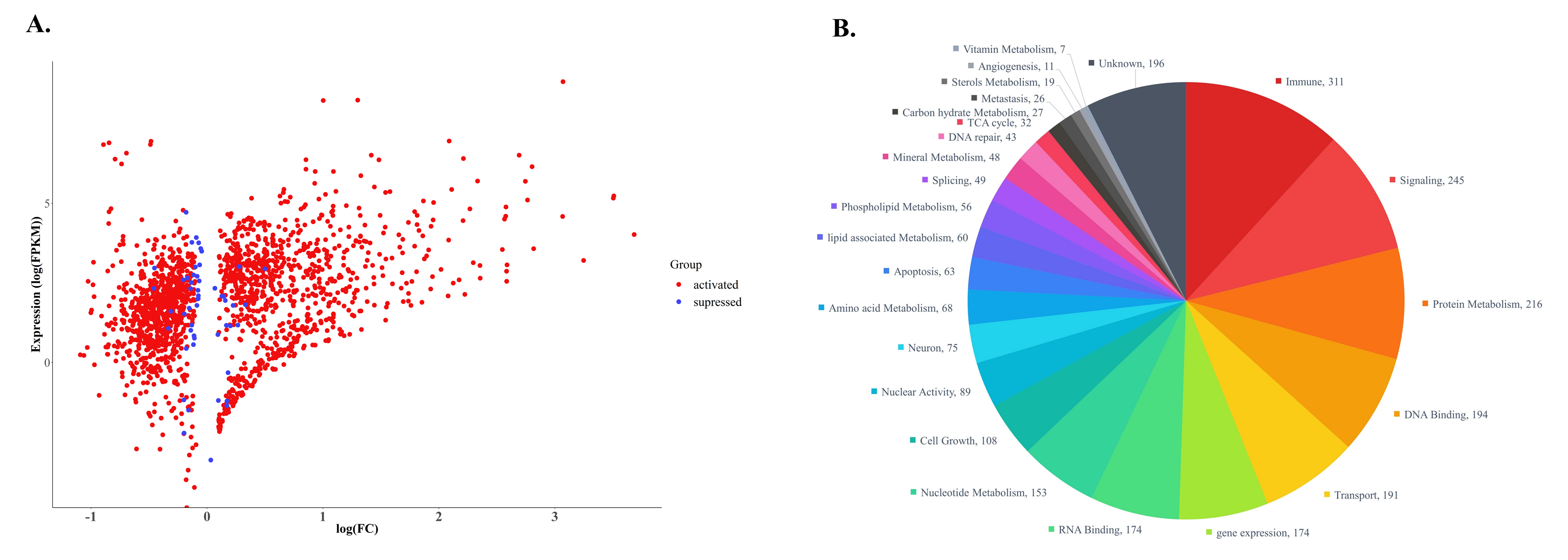
**

**Additional file 6. RNA-seq analysis of the immunomodulation activities by LPPC/MP complex with different antibodies.**

**(A)** Volcano plot for gene expression and the FC values of genes. **(B)** Pie chart demonstrating the proportion of mouse RNA-seq reads assigned to annotated genomic functions.
