# Supplementary material for: A flexible liposomal polymer complex as a platform of specific and regulable immune regulation for individual cancer immunotherapy
Source: J Exp Clin Cancer Res. 2023 Jan 23;42:29. doi: 10.1186/s13046-023-02601-8 (PMC9869520; doi:10.1186/s13046-023-02601-8)
Supplement: Supplementary file 7 — Additional file 7. RNA-seq analysis of the immunomodulation activitiesby LPPC/MP complex with different antibodies. The RNA expression levels of immunecells under different treatments were determined by t-tests and ANOVA,and the results were shownas boxplots. The description of group names was the same as in Fig. 9. [file 13046_2023_2601_MOESM7_ESM.docx]

**
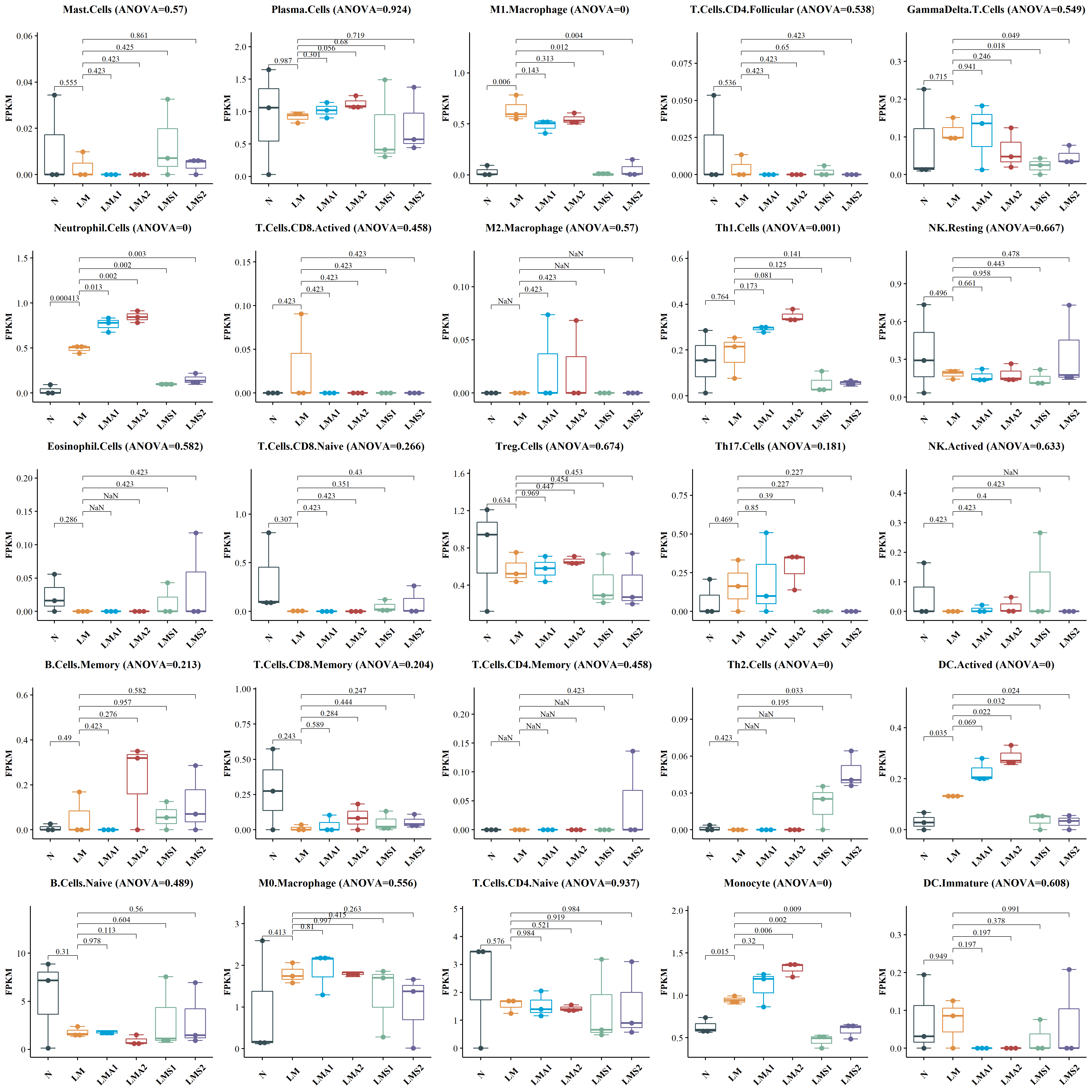
**

**Additional file 7. RNA-seq analysis of the immunomodulation activities by LPPC/MP complex with different antibodies.**

The RNA expression levels of immune cells under different treatments were determined by *t*-tests and ANOVA, and the results were shown as boxplots. The description of group names was the same as in Fig. 9.
